# Integrating Smart Health in the US Health Care System: Infodemiology Study of Asthma Monitoring in the Google Era

**DOI:** 10.2196/publichealth.8726

**Published:** 2018-03-12

**Authors:** Amaryllis Mavragani, Alexia Sampri, Karla Sypsa, Konstantinos P Tsagarakis

**Affiliations:** ^1^ Department of Computing Science and Mathematics Faculty of Natural Sciences University of Stirling Stirling United Kingdom; ^2^ Department of Pharmacy and Forensic Science King's College London University of London London United Kingdom; ^3^ Business and Environmental Technology Economics Lab Department of Environmental Engineering Democritus University of Thrace Xanthi Greece

**Keywords:** asthma, big data, forecasting, Google trends, health care, health informatics, internet behavior, nowcasting, online behavior, smart health

## Abstract

**Background:**

With the internet’s penetration and use constantly expanding, this vast amount of information can be employed in order to better assess issues in the US health care system. Google Trends, a popular tool in big data analytics, has been widely used in the past to examine interest in various medical and health-related topics and has shown great potential in forecastings, predictions, and nowcastings. As empirical relationships between online queries and human behavior have been shown to exist, a new opportunity to explore the behavior toward asthma—a common respiratory disease—is present.

**Objective:**

This study aimed at forecasting the online behavior toward asthma and examined the correlations between queries and reported cases in order to explore the possibility of nowcasting asthma prevalence in the United States using online search traffic data.

**Methods:**

Applying Holt-Winters exponential smoothing to Google Trends time series from 2004 to 2015 for the term “asthma,” forecasts for online queries at state and national levels are estimated from 2016 to 2020 and validated against available Google query data from January 2016 to June 2017. Correlations among yearly Google queries and between Google queries and reported asthma cases are examined.

**Results:**

Our analysis shows that search queries exhibit seasonality within each year and the relationships between each 2 years’ queries are statistically significant (*P*<.05). Estimated forecasting models for a 5-year period (2016 through 2020) for Google queries are robust and validated against available data from January 2016 to June 2017. Significant correlations were found between (1) online queries and National Health Interview Survey lifetime asthma (*r*=–.82, *P*=.001) and current asthma (*r*=–.77, *P=*.004) rates from 2004 to 2015 and (2) between online queries and Behavioral Risk Factor Surveillance System lifetime (*r*=–.78, *P*=.003) and current asthma (*r*=–.79, *P*=.002) rates from 2004 to 2014. The correlations are negative, but lag analysis to identify the period of response cannot be employed until short-interval data on asthma prevalence are made available.

**Conclusions:**

Online behavior toward asthma can be accurately predicted, and significant correlations between online queries and reported cases exist. This method of forecasting Google queries can be used by health care officials to nowcast asthma prevalence by city, state, or nationally, subject to future availability of daily, weekly, or monthly data on reported cases. This method could therefore be used for improved monitoring and assessment of the needs surrounding the current population of patients with asthma.

## Introduction

Health informatics is the field where information technology, computer science, social sciences, and health care meet [1]. Recently, with the use of big data (ie, large data volumes characterized by high speed and wide dataset variety [2-4]) being all the more applied in research in general, health informatics provides fertile ground for big data applications.

According to Gu et al [5], big data health care research consists of 3 research stages: disease, life and health, and nursing. Focus is being given to various aspects of diseases, technology, and health care services in areas such as epidemics, data mining, machine learning, and customized service [5]. Big data is being increasingly integrated in health care informatics [5-6] and has been used in the past in smart city management.

Over the last few years during the integration of the health pillar in smart cities, where big data is being continuously gathered and analyzed [7], the concept of smart health has been rising [8-10]. Smart health as a concept is derived from the intersection of medical informatics, public health, and business, where large volumes of social media data, payer-provider big data, genomic-driven big data, and biomedical data are being used for the monitoring and evaluation of patients’ conditions [10]. As life expectancy increases, so does the cost of health care, and thus innovative methods are required to achieve improved cost-effective quality services. The use of big data in smart health can assist in P4 medicine (preventive, participatory, predictive, and personalized) [8], in the detection, prediction, and prevention of diseases [5], and in the health industry in general [10] while also taking into account the cost, data sources and quality, and population [4].

What has been of notable popularity in big data analytics is the analysis of online search queries [11-12], mainly using Google Trends [13], a popular open tool that has been widely integrated in scientific research over the course of the past decade, mainly focused on health-related topics [6]. Examples include analysis of online interest in multiple sclerosis [14], epilepsy [15-16], silicosis [17], dementia [18], urinary tract infection [19], Ebola [20], the flu [21-23], tobacco and lung cancer [24], epidemics [25-26], and even in illegal drugs such as dabbing [27], krokodil [28], and methamphetamine [29]. This use of big data has formed the cornerstone of a new concept, the science of infodemiology, which uses the vast variety of data available on the internet such as online queries, publications, or posts on blogs and websites for real-time data analysis with the aim of informing public health and public policy, thus providing a viable alternative to the time-consuming traditional methods of gathering health care data such as population surveys and registries. The use of infodemiology data for surveillance purposes is called infoveillance and could potentially allow for more timely and targeted health care interventions [30].

In this study, online queries for the term “asthma” in the United States were analyzed in order to explore the possibility of nowcasting (ie, forecasting the present) asthma prevalence using Google Trends. Asthma was selected because it is a common chronic respiratory disease characterized by exacerbations, also known as asthma attacks; therefore, the reported cases are bound to show seasonality as well as constant interest.

Asthma is a chronic condition characterized by airway inflammation and hyper-responsiveness that causes airways to constrict in response to exercise, infection, exposure to allergens, and occupational exposures [31]. In 2014, it was estimated that approximately 7.4% of the adult US population and 8.6% of US children lived with asthma [32]. During childhood, asthma is more prevalent in males, whereas in adulthood prevalence shifts toward females. Black and multirace people also have a higher prevalence than white people [33-34].

Asthma presents with coughing, wheezing, and chest tightness that seem to be worse during the night and early mornings. These symptoms, along with a family history of asthma or atopic dermatitis, can prompt investigations to confirm an asthma diagnosis. Exacerbation of normal asthma symptoms is more common in patients with uncontrolled asthma or in high-risk patients [35]. Certain types of asthma exacerbations are linked to particular seasons of the year with those caused by pollen and mold being truly seasonal [36]. It has been shown that pediatric patients experience a peak of asthma exacerbations during the fall and spring months [37], whereas adult patients experience a peak of asthma exacerbations at year end [38].

The management of asthma usually involves the use of several inhalers, leading to a rather complicated treatment regime that presents difficulties in terms of patient compliance because it interferes with their daily living activities. Poor compliance can lead to increased morbidity as well as increased cost of treatment [39]. Apart from treatment compliance, another important factor that weighs in the success of the treatment is inhaler technique, as improper inhaler use is linked to poor asthma control. Studies have shown that 33% to 94% of patients do not receive any training regarding proper inhaler technique, which leads to a great number of patients using inhalers incorrectly [40]. Asthma self-management education and personalized advice can improve a patient’s asthma control and quality of life, along with reducing asthma exacerbations and hospital admissions [41].

Asthma has several social complications such as limiting patients’ activity levels [42], which has an economic impact on the country’s health care system. It was estimated that in 2007, medical expenses, missed work and school days, and early deaths due to asthma cost the United States $56 billion [43].

Google Trends data have been previously shown to be valid by many studies [44], and work on the subject has shown the tool’s contribution to forecasting [45-46] and analysis of online behavior, provided careful selection of the examined terms [47]. The aim of this paper is to examine if nowcasting asthma prevalence in the United States is possible using online search traffic data.

## Methods

Monthly time series from Google Trends for the keyword “asthma” from 2004 to 2015 in the United States and by individual state were used. The data were normalized by Google and downloaded in .csv format on July 7, 2017, between 12:47 and 13:02 for the United States and on July 18 between 14:03 and 14:33 for each of the 50 states and the District of Columbia. The data adjustment procedure is reported by Google as follows [48]: “Search results are proportionate to the time and location of a query: Each data point is divided by the total searches of the geography and time range it represents, to compare relative popularity. Otherwise places with the most search volume would always be ranked highest. The resulting numbers are then scaled on a range of 0 to 100 based on a topic’s proportion to all searches on all topics. Different regions that show the same number of searches for a term will not always have the same total search volumes.”

**Figure 1:**
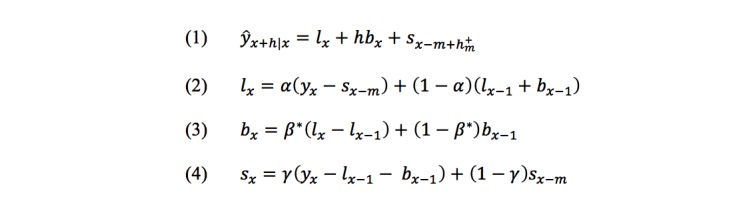
Equations for Holt-Winters exponential smoothing, where *y*_*x*_ and *ŷ*_*x*_ denote the initial series and the forecasts, respectively. The *l*_*x*_, *b*_*x*_, and *s*_*x*_ denote the level, the trend, and seasonal estimates for month *x*, respectively, with *m* denoting the period of the seasonality (ie, 12 in this case), and *h*^+^_*m*_=⌊(*h*–1)*mod m*⌋+1. The level, trend, and seasonal change smoothing factors are denoted by constants *α*, β*, and *γ*, respectively. The estimated values for the coefficients for the level and trend are denoted by *a* and *b*, respectively, while the seasonal coefficients are denoted by *s*_1_,...,*s*_12_, for month 1,...,12, respectively.

The seasonality of asthma queries was explored followed by the estimation of the forecasts for the online interest in the term from 2016 through 2020 for the country as well as for each state. The additive method for the Holt-Winters exponential smoothing (using the statistical programming language R) is employed. The Holt-Winters equations [49] can be seen in Figure 1.

In order to further elaborate on the seasonality, the Pearson correlations for Google Trends data for the term “asthma” between each 2 years from 2004 to 2015 in the United States were calculated. Finally, the Pearson correlations between Google queries and the National Health Interview Survey (NHIS) prevalence data [50] from 2004 to 2015 and Behavioral Risk Factor Surveillance System (BRFSS) prevalence data [51] from 2004 to 2014 were examined.

Asthma is not included in the list of diseases with a Centers for Disease Control and Prevention (CDC) surveillance case definition, defined as “a set of uniform criteria used to define a disease for public health surveillance. Surveillance case definitions enable public health officials to classify and count cases consistently across reporting jurisdictions. They provide uniform criteria of national notifiable infectious and non-infectious conditions for reporting purposes” [52]. Thus, nationwide surveys are used to gather information regarding asthma prevalence, including additional information on asthma control, medications, and hospitalizations [53]. The BRFSS is a “state-based, random-digit–dialed telephone survey designed to monitor the prevalence of the major behavioral risks among adults associated with premature morbidity and mortality,” and the NHIS is a “multistage probability sample survey designed to solicit health and demographic information about the population, conducted annually with face-to-face interviews in a nationally representative sample of households” [54].

In 2011, the BRFSS changed its weighting methodology in addition to also including mobile phone respondents. Therefore, any comparisons between years before and after 2011 should be carefully interpreted. In this study, no such comparisons are made, as each year’s online queries are compared with the respective year’s asthma reported cases, thus including no cross-year comparisons. For this study, we used the CDC definition of asthma prevalence, based on affirmative responses to the following NHIS questions: (adults) “Have you ever been told by a doctor or other health professional that you had asthma?” and “Do you still have asthma?” and (children) “Has a doctor or other professional ever told you that [sample child] had asthma?” and “Does [sample child] still have asthma?” [55].

## Results

### Online Interest in the United States

**Figure 2:**
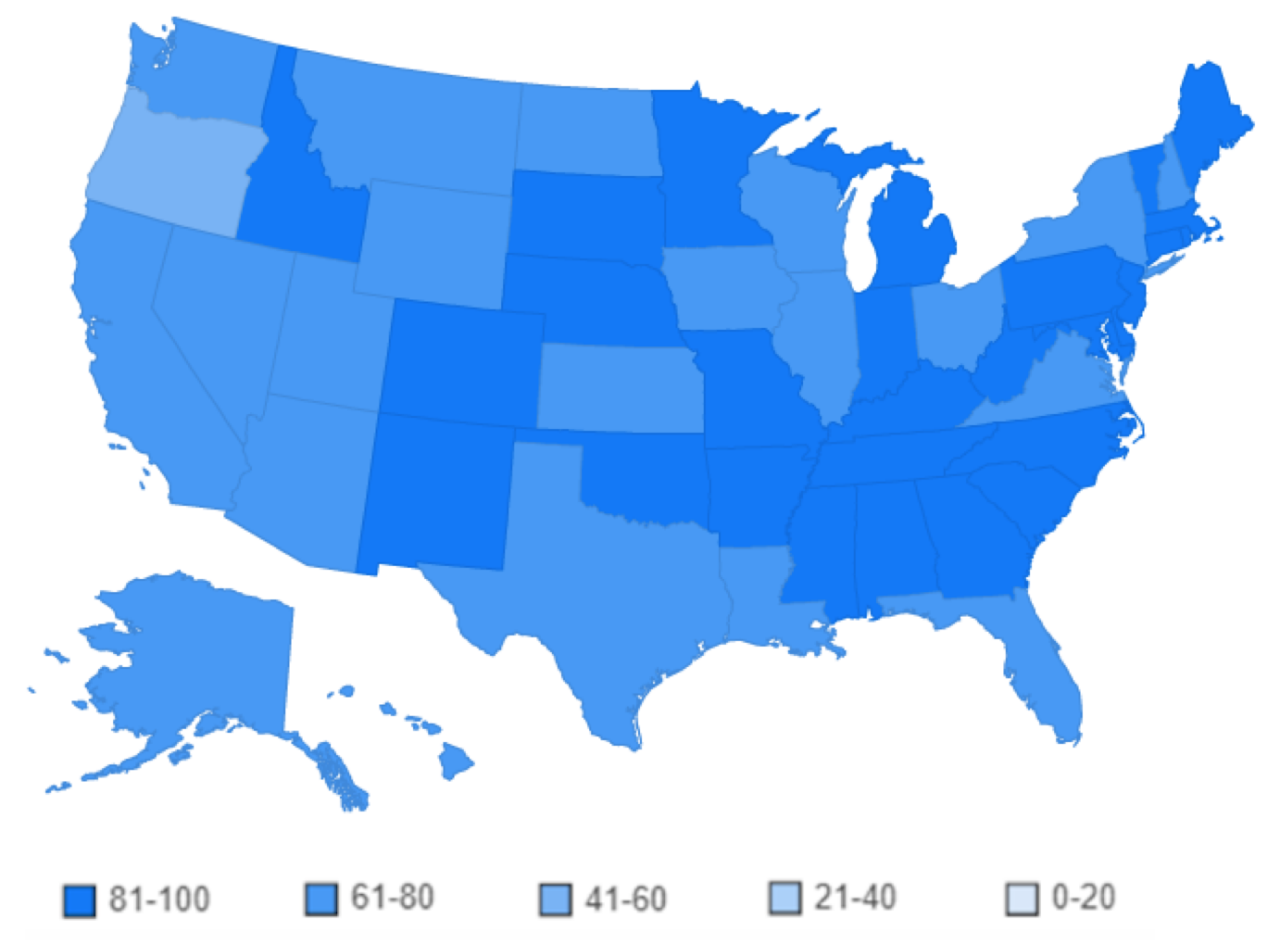
Online interest by state in the term "asthma" from 2004 to 2015.

Figure 2 shows a heat map of the United States classified into 5 groups of interest in the term “asthma” from 2004 to 2015 (ie, 0 to 20, 21 to 40, 41 to 60, 61 to 80, and 81 to 100; light blue to darker blue).

Out of the 50 states and District of Columbia, 29 fall into the 81 to 100 group, 21 in the 61 to 80 group, only 1 (Oregon) in the 41 to 60 group, and none in the 21 to 40 and 0 to 20 groups. This classification indicates that the examined term is of high interest to the population of the United States. The detailed data for Figure 2 are available in Multimedia Appendix 1, Table A1.

**Figure 3:**
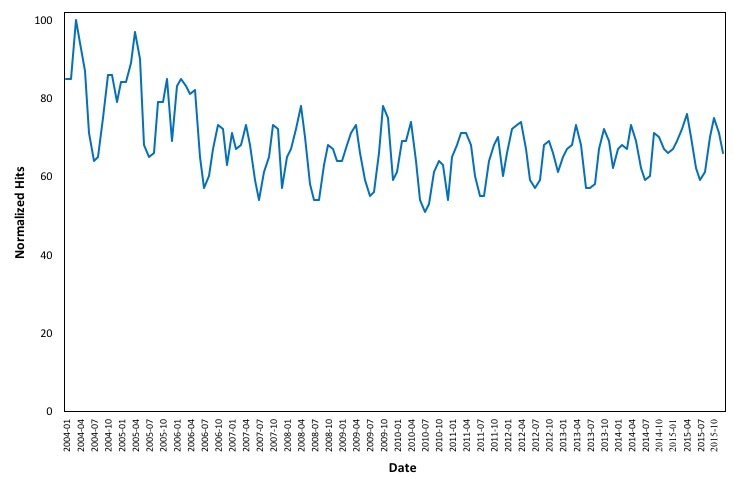
Monthly changes in online interest in the term "asthma" from 2004 to 2015.

**Figure 4:**
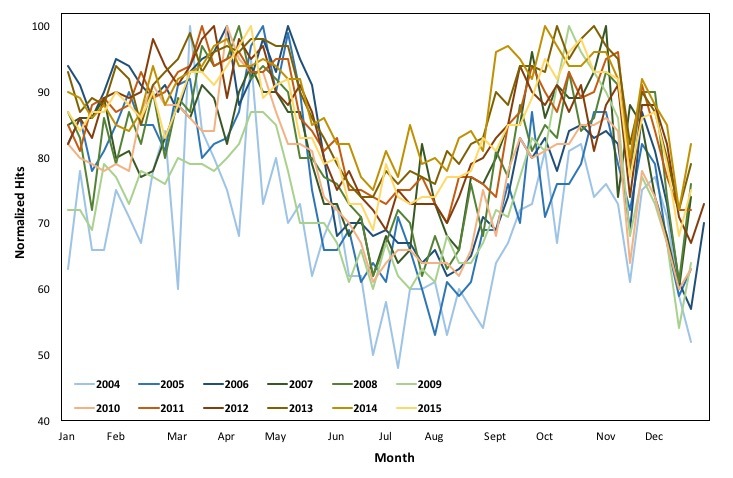
Weekly changes in online interest in the term "asthma" for each year from 2004 to 2015.

Figures 3 and 4 depict the changes in online interest in the term “asthma” for the period 2004 to 2015 and the seasonal changes for each year from 2004 to 2015, respectively. As is evident, the data follow a seasonal trend. All years’ data, as presented in Figure 4, follow a similar pattern during a full year, supporting our hypothesis that the seasonality of asthma prevalence in the United States is depicted in online searches.

**Figure 5:**
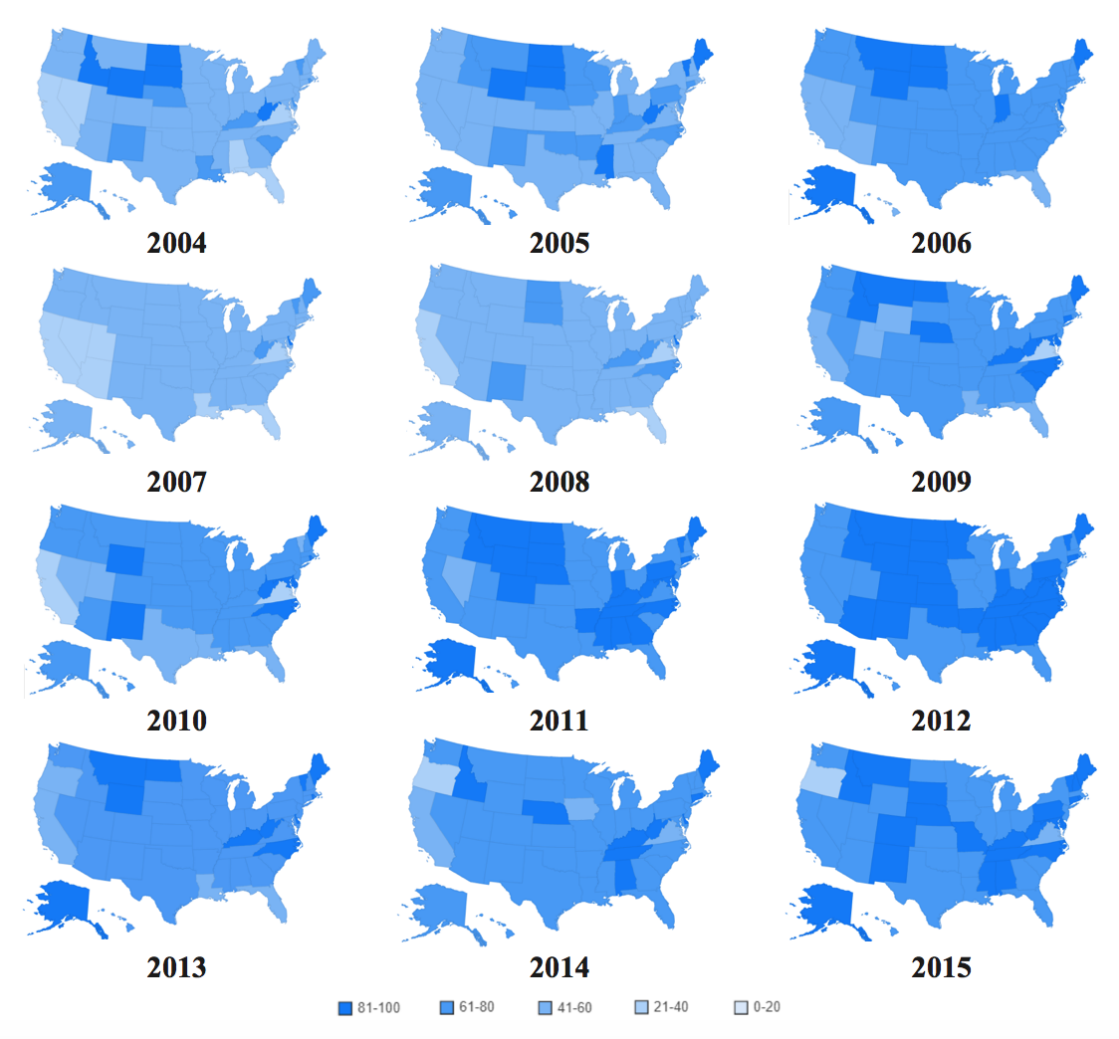
Online interest by state in the term "asthma" per year from 2004 to 2015.

Figure 5 consists of the changes by state in online interest in the term “asthma” by year from 2004 to 2015. All data are available in Multimedia Appendix 1, Table A2.

There has been a significant increase in searches for the term “asthma” in the states from 2004 to 2015, with the lowest count of states in the 81 to 100 group being in 2007 and the highest in 2012. The top asthma-related queries in the United States from January 2004 to December 2015 include “allergy asthma” (100), “asthma symptoms” (45), “asthma attack” (35), “what is asthma” (25), “asthma inhaler” (20), “asthma children” (15), “exercise asthma” (15), “asthma medications” (10), and “allergy and asthma center” (10).

As is evident, online behavioral changes toward the term “asthma” depict behavior toward said disease. The next steps are to examine if forecasting online interest in the United States is possible and identify existing relationships between online search traffic data and reported asthma cases.

### Forecasting Online Interest in the United States

**Figure 6:**
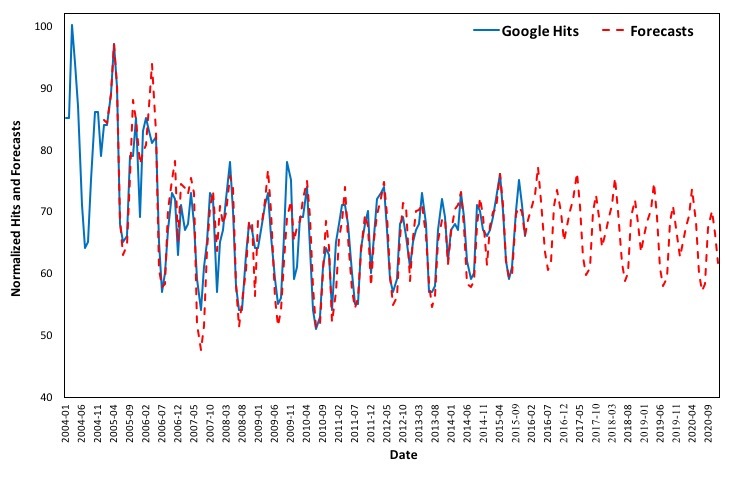
Google Trends (2004 to 2015) versus forecasts (2005 to 2020) in the United States.

Figure 6 depicts changes in online interest over the period 2004 to 2015 and estimated forecasts from 2005 to 2020. The estimated model closely approximates the actual Google queries for the term “asthma” in the United States over the examined period.

The smoothing parameters for the additive Holt-Winters exponential smoothing with trend and additive seasonal component are α=.33, β*=0, and γ=.65. The estimated values for the coefficients for the level, trend and season are as follows: a=69.54, b=–.07, s_1_=–.94, s_2_=1.44, s_3_=3.37, s_4_=7.84, s_5_=2.51, s_6_=–5.68, s_7_=–8.51, s_8_=–7.20, s_9_=1.89, s_10_=4.67, s_11_=1.11, and s_12_=–3.53.

**Figure 7:**
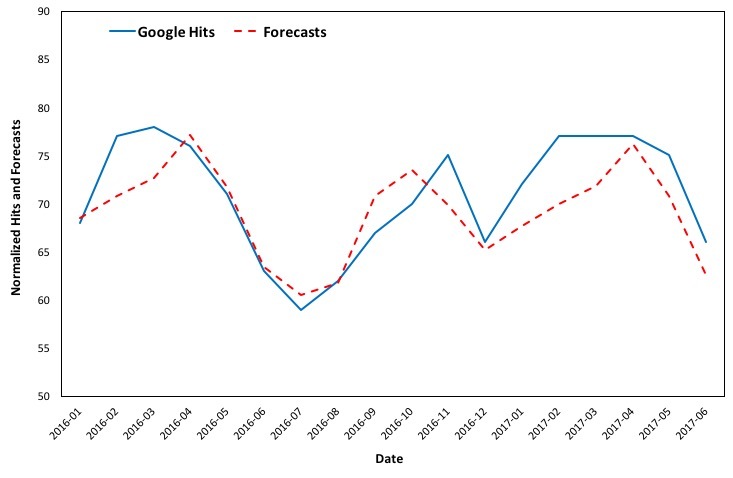
Google Trends (2004 to 2015) versus forecasts (January 2016 to June 2017) in the United States.

In order to elaborate on the robustness of the forecasting model, the estimated values are validated against the available Google queries for the term “asthma” from January 2016 to June 2017, as is shown in Figure 7. It is evident that the forecasts follow the same curve and well approximate the actual Google Trends data for the aforementioned period.

It is therefore suggested that the online behavior exhibits seasonality and can be predicted. The last step in exploring if nowcasting of asthma prevalence in the United States is possible using Google Trends is to examine the correlations between Google Trends data and reported lifetime and current asthma.

### Google Trends Versus Reported Asthma

As shown in Figure 4, each examined year’s online interest seems to follow a similar seasonal trend from January to December. To elaborate on the seasonal trend, the Pearson correlations between each 2 years’ queries are calculated (Table 1). The monthly Google Trends data between each 2 years from 2004 to 2015 exhibit high correlations, while all comparisons are statistically significant, with *P*<.05.

**Table 1:** Pearson correlations between each 2 years’ normalized Google asthma queries in the United States from 2004 to 2015.

	2004	2005	2006	2007	2008	2009	2010	2011	2012	2013	2014
2005	.89	—	—	—	—	—	—	—	—	—	—
2006	.86	.89	—	—	—	—	—	—	—	—	—
2007	.77	.85	.77	—	—	—	—	—	—	—	—
2008	.94	.93	.81	.78	—	—	—	—	—	—	—
2009	.79	.76	.64	.89	.80	—	—	—	—	—	—
2010	.88	.94	.87	.82	.92	.81	—	—	—	—	—
2011	.94	.93	.85	.87	.93	.91	.93	—	—	—	—
2012	.88	.90	.85	.81	.90	.82	.98	.91	—	—	—
2013	.84	.87	.72	.89	.90	.93	.89	.92	.90	—	—
2014	.75	.82	.68	.77	.87	.78	.82	.83	.86	.92	—
2015	.86	.85	.69	.86	.92	.93	.88	.92	.90	.98	.93

**Table 2:** Total lifetime and current asthma National Health Interview Survey (2004 to 2015) and Behavioral Risk Factor Surveillance System (2004 to 2014) prevalence data.

Year	NHIS^a^			BRFSS^b^		
	Lifetime asthma	Current asthma	Asthma hits^c^	Lifetime asthma	Current asthma	Asthma hits^c^
2004	30,189	20,545	81.41	33,084,183	20,422,385	83.17
2005	32,621	22,227	79.58	30,661,476	19,453,974	80.33
2006	34,132	22,876	72.58	35,107,599	22,853,570	73.92
2007	34,008	22,879	65.66	36,832,798	23,556,048	68.17
2008	38,450	23,333	65.00	38,050,505	24,521,005	66.92
2009	39,930	24,567	65.83	38,033,371	24,051,245	67.92
2010	39,191	25,710	61.41	39,005,338	25,069,373	62.83
2011	39,504	25,943	64.58	34,759,106	22,605,961	66.42
2012	39,982	25,553	65.91	39,085,744	25,954,771	67.67
2013	37,328	22,648	65.25	41,030,777	26,227,484	67.00
2014	40,461	24,009	66.58	40,706,401	26,957,918	68.75
2015	40,153	24,633	68.16	—	—	—

^a^NHIS: National Health Interview Survey.

^b^BRFSS: Behavioral Risk Factor Surveillance System.

^c^Values slightly vary due to the different time frame: 2004 to 2015 for NHIS and 2004 to 2014 for BRFSS.

To further explore the relationships between online searches and asthma prevalence in the United States, data on the yearly cases of lifetime and current asthma for all ages from the NHIS prevalence data from 2004 to 2015 [50] and the BRFSS prevalence data [51] from 2004 to 2014 (Table 2) are used.

The Pearson correlations of the annual NHIS prevalence data with the annual averages of the normalized Google Trends data from 2004 to 2015 show high correlations between lifetime asthma (*r*=–.82, *P*=.001) and current asthma (*r*=–.77, *P=*.004). BRFSS prevalence data also exhibit high correlations with Google Trends data for lifetime (*r*=–.78, *P*=.003) and current asthma (*r*=–.79, *P*=.002). The Spearman correlations for the aforementioned pairs of variables all exhibit the same negative relationship, although not all are statistically significant.

Although statistically significant, all Pearson correlations are negative, and lag analysis should be employed to identify the time interval of response between asthma online interest and case reporting or vice versa. Although Google Trends data for the term “asthma” in the United States over the examined period are monthly, the data on lifetime and current asthma are yearly; until weekly or monthly data are available, further analysis cannot by done.

### Forecasting Online Interest by State

In order to show that the method of nowcasting asthma prevalence in the United States using Google queries is possible, this methodology is applied in each of the 50 states and the District of Columbia and exhibits good forecasting results. Figures 8 to 11 depict the changes in online interest in the term “asthma” from 2004 to 2015 and forecasts from 2016 to 2020 for the 4 most populated states (ie, California, Texas, Florida, and New York), and the graphs for all states can be found in Multimedia Appendix 2, Figures B1-B51. The values of the smoothing parameters *α*, β*, and *γ* and the coefficients for each state’s forecasts can be found in Multimedia Appendix 1, Tables A3 and A4, respectively. As online behavioral changes can be predicted and data on asthma cases are correlated with online queries, nowcasting of asthma could be possible provided short-interval data (eg, monthly, weekly, or even daily) are available.

According to the results, online interest in Alaska, Nebraska, New Hampshire, Oklahoma, and Tennessee exhibits increasing forecast trends from 2016 to 2020. On the contrary, online interest in Delaware, Kansas, Oregon, and Virginia exhibits decreasing forecast trends from 2016 to 2020. Overall, the states of Arizona, California, Connecticut, Florida, Georgia, Illinois, Indiana, Maryland, Michigan, Missouri, New Jersey, New York, North Carolina, Pennsylvania, Texas, and Washington show high interest in the term “asthma” throughout the examined period, while in Hawaii and Wyoming, interest is low. Virginia is the only state where online interest exhibits very significant variations from 2004 to 2016.

Our study indicates that analysis of online behavior toward asthma by state can assist with nowcasting asthma prevalence. Since search queries and reporting of asthma are shown to correlate in the United States, if short-interval data (eg, weekly or monthly) were made available, a robust nowcasting model could be developed.

**Figure 8:**
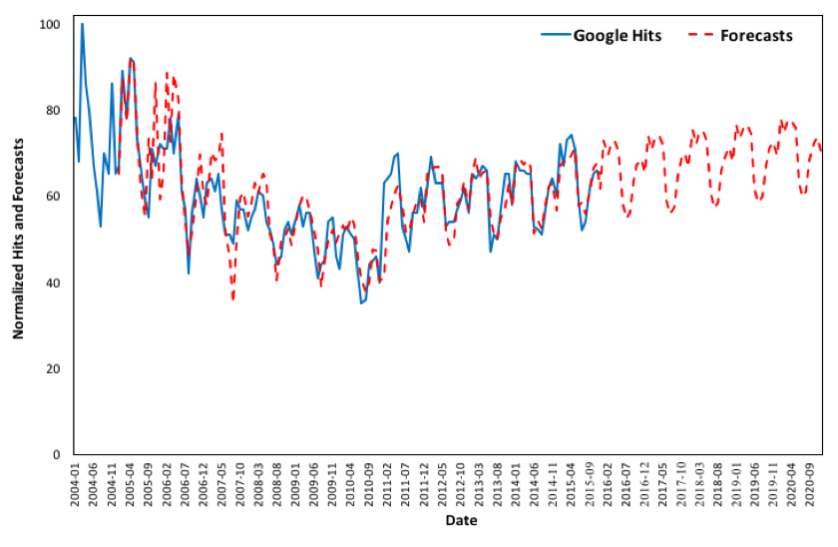
Google Trends (2004 to 2015) versus forecasts (2005 to 2020) in California.

**Figure 9:**
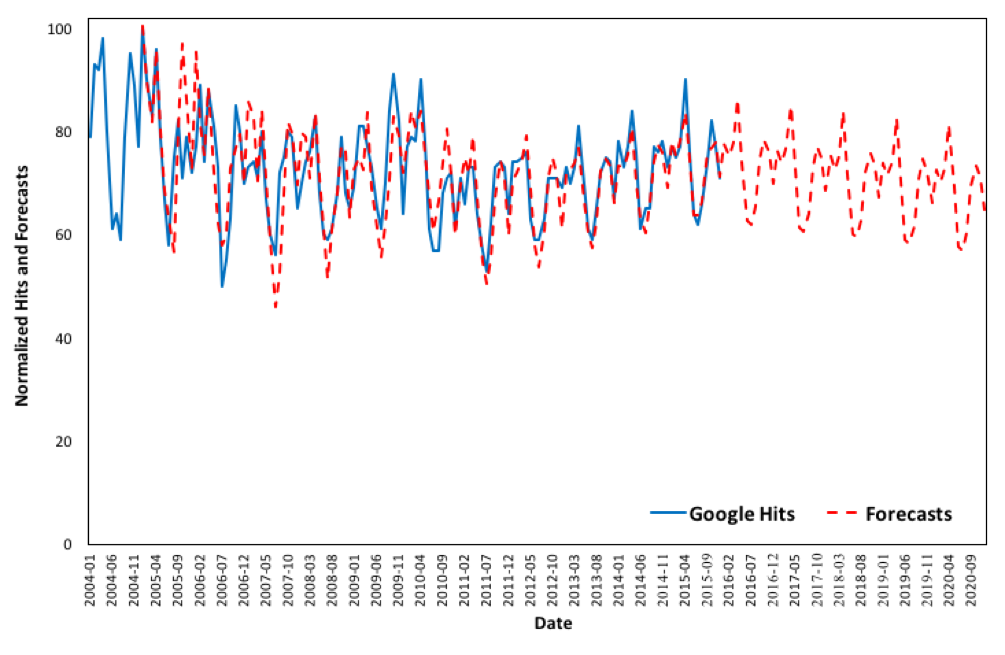
Google Trends (2004 to 2015) versus forecasts (2005 to 2020) in Texas.

**Figure 10:**
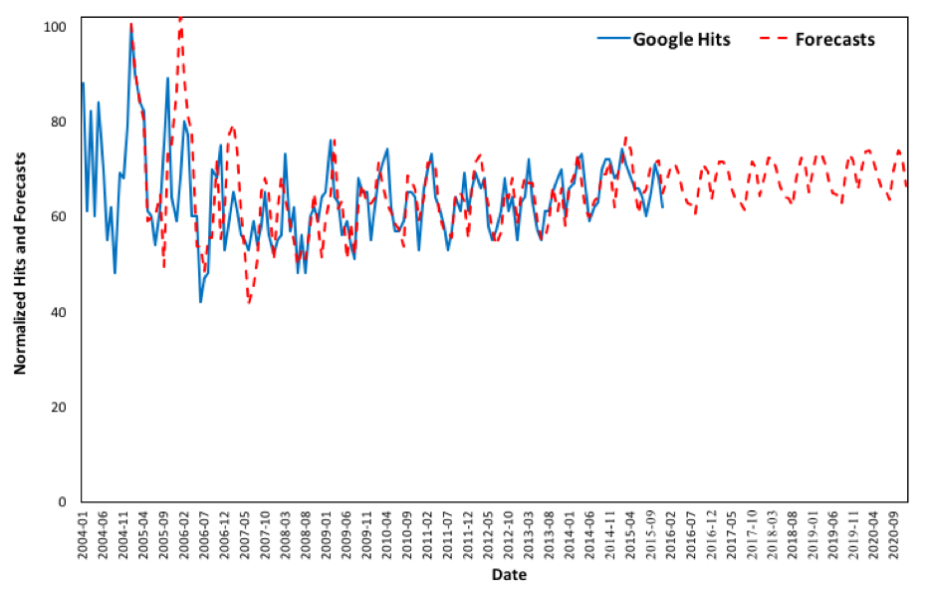
Google Trends (2004 to 2015) versus forecasts (2005 to 2020) in Florida.

**Figure 11:**
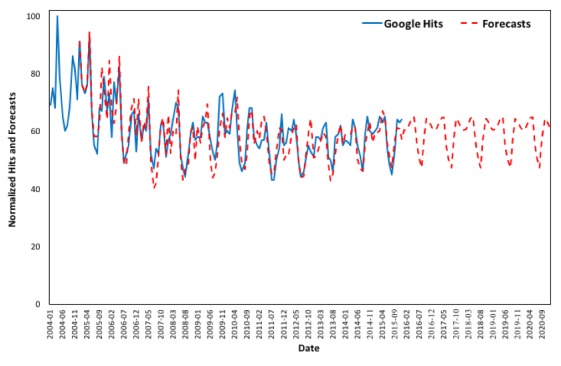
Google Trends (2004 to 2015) versus forecasts (2005 to 2020) in New York.

## Discussion

### Principal Findings

In addressing integration of smart health into smart city management, monitoring of search traffic data could be useful in predictions and nowcastings, as has also been suggested by previous work on the subject. This study shows that online interest can be predicted nationally and by state. Therefore, governments, policy makers, and health care officials have the ability to use these data to better address the responsiveness of the US health care system at national, regional, state, or even city level in order to nowcast asthma prevalence. Google Trends also provides detailed regional US data, and this method can be applied in other countries as well.

Empirical relationships between Google Trends and human behavior have been suggested, therefore nowcasting asthma prevalence in the United States is possible using online search traffic data, subject to availability of daily, weekly, or monthly data. In this study, it was shown that online search traffic data are highly correlated between each 2 years during the examined period and that Google Trends data are correlated with reported cases of lifetime and current asthma in the United States from 2004 to 2015.

After analyzing changes in online interest in the United States over the examined period, the next step was to identify any seasonal similarities between each 2 years’ (monthly) search queries. As the hits between each 2 years from 2004 to 2015 on the term “asthma” were highly correlated, the seasonal effect was evident; using Holt-Winters exponential smoothing, 5-year forecasts for online interest in the term from 2016 to 2020 nationally and in each state were estimated. Validated against available data from January 2016 to June 2017, the forecasts were well fitted and accurately approximated the actual Google Trends data for the same period, suggesting seasonal behavioral changes over the course of a year can be accurately predicted using the proposed method. Google Trends data are correlated with reported cases of lifetime and current asthma, and thus nowcasting asthma prevalence in the United States is suggested to be possible using online search traffic data. As the calculated correlations are negative at this point and there is a lag between internet queries and asthma reporting and vice versa, short-interval data (eg, monthly, weekly, and daily—not available at this point) are required in order to identify said lag.

### Limitations

This study has limitations. It cannot be assumed that each hit corresponds to an asthma case and vice versa because hits could be also attributed to academic or research reasons or general interest on the subject, and they could be influenced by news reports or social media. Queries related to asthma could be also influenced by factors such as changes of health insurance and weather or environmental conditions that trigger similar symptoms. This is a general limitation when examining online queries, despite the empirical relationships that have been shown to exist between Google Trends and health data.

The sample is not representative, although as internet penetration increases, so does the possibility of higher volumes of online queries being related to asthma cases. Additionally, nowcasting asthma prevalence using online search queries is not possible at this point because the available data on reported lifetime and current asthma are yearly. If monthly, weekly, or daily data on past asthma prevalence were available and the correlations between search traffic data and reported asthma are validated, the possibility of nowcasting asthma could be further explored.

This study has not accounted for state-by-state confounders that could influence search patterns, such as the socioeconomic status and demographics of different states that might be relevant to asthma prevalence, as this exceeds the scope of this paper. The latter, along with the impact of socioeconomic and cultural differences on asthma reporting and online search patterns, are of interest for further investigation. In addition, more search terms related to asthma symptoms such as “breathlessness” and “wheezing” could be included in future research on asthma monitoring in the United States.

### Conclusion

The findings of this study support previous work on the subject and highlight the value of online data in health and medical informatics. Google Trends data have been shown to be useful and valuable in the monitoring, surveillance, or prediction of epidemics and outbreaks [20,25-26,56], as have been various other internet sources such as Twitter [57], medical portals [58], and Baidu [59]. Google queries provide us with the revealed and not the stated user interest contrary to traditional survey methods [60], and the use of Web data will benefit the exploration of behavior in medical issues [61]. Data from traditional sources and big data should be combined in order to take full advantage of all available information [62]. When daily, weekly, or monthly data on reported asthma cases are made available, data from online sources like Google Trends could be used centrally and then applied by state or used by each city or state individually, assisting with the integration of the smart health concept in smart city management.

Internet behavior can be measured by infodemiology metrics as information patterns and population health are related [30]. Surveillance of asthma is mainly assessed through nationwide surveys and interviews, and data on asthma prevalence are only available long after the cases of asthma are reported. Nowcasting Google queries on selected terms related to asthma could assist health officials at both national and state levels to detect any behavioral variations toward the disease, providing time-effective allocation of resources and a more cost-effective approach to asthma assessment. This study suggests a relationship between asthma prevalence and Google Trends data. In the future, analysis of online queries could be valuable in the monitoring and evaluation of the responsiveness of the US health care system to asthma patient admissions and prescription drug needs, as well as assisting with the implementation of targeted health interventions and campaigns during periods when increased asthma admissions are predicted.

## Appendix

Multimedia Appendix 1 State data tables.

Multimedia Appendix 2 Google Trends (2004 to 2015) versus forecasts (2005 to 2020) by state.

## Abbreviations

BRFSS: Behavioral Risk Factor Surveillance System
CDC: Centers for Disease Control and Prevention
NHIS: National Health Interview Survey
