# Supplementary material for: Integrating Smart Health in the US Health Care System: Infodemiology Study of Asthma Monitoring in the Google Era
Source: JMIR Public Health Surveill. 2018 Mar 12;4(1):e24. doi: 10.2196/publichealth.8726 (PMC5869181; doi:10.2196/publichealth.8726)
Supplement: Multimedia Appendix 1 [file publichealth_v4i1e24_app1.pdf]

Multimedia Appendix 1: State data tables

Table A1 consists of the States by declining interest in the term ‘Asthma’ from 2004 to 2015, Table A2 consists of the normalized values for the online interest by State for each year from 2004 to 2015, and Tables A3 and A4 present the smoothing parameters and coefficients for the Holt-Winters forecasting by State, respectively.

Table A1. Online Interest for ‘Asthma’ by State in USA from January 2004 to December 2015

State	Score	State	Score	State	Score	State	Score
Delaware	100	South Carolina	89	Massachusetts	81	Arizona	78
West Virginia	97	Indiana	88	Oklahoma	81	Wisconsin	77
North Carolina	95	Colorado	87	New Jersey	81	Texas	76
Kentucky	95	Alabama	87	Montana	80	Hawaii	73
Maine	94	Georgia	86	Washington	80	Utah	72
Tennessee	93	South Dakota	86	New York	80	Iowa	72
Connecticut	92	New Mexico	85	Kansas	79	Louisiana	71
Maryland	92	Vermont	85	Alaska	79	Florida	69
Mississippi	92	Missouri	85	New Hampshire	78	Nevada	67
Pennsylvania	91	Minnesota	84	Ohio	78	California	67
Nebraska	91	Arkansas	84	North Dakota	78	Virginia	61
Idaho	89	District of Columbia	83	Wyoming	78	Oregon	55
Rhode Island	89	Michigan	83	Illinois	78		

Table A2. Online Interest for 'Asthma' by State by Year from 2004 to 2015

State	2004	2005	2006	2007	2008	2009	2010	2011	2012	2013	2014	2015
**Alabama**	38	58	76	50	52	73	80	83	90	77	82	88
**Alaska**	67	73	100	52	44	77	70	88	86	86	79	93
**Arizona**	48	52	58	39	47	67	64	72	83	66	70	78
**Arkansas**	45	76	80	51	54	63	74	85	82	80	78	79
**California**	40	45	53	35	36	44	37	61	65	56	57	63
**Colorado**	48	54	62	52	53	73	75	83	87	73	72	81
**Connecticut**	54	63	76	48	59	81	75	93	91	78	87	90
**Delaware**	78	79	95	100	100	100	82	93	95	100	100	100
**DC**	52	52	68	43	45	68	64	78	82	71	74	78
**Florida**	37	41	49	34	40	57	55	65	68	56	61	65
**Georgia**	48	54	67	50	51	73	68	83	89	73	71	79
**Hawaii**	58	62	52	43	48	63	67	71	80	62	64	66
**Idaho**	81	73	65	52	58	89	77	95	97	80	87	85
**Illinois**	52	56	67	42	49	69	64	69	75	64	66	72
**Indiana**	59	69	82	45	51	77	71	81	88	70	76	78
**Iowa**	55	62	68	43	47	75	73	73	75	61	60	67
**Kansas**	51	57	68	47	48	64	77	79	85	69	67	62
**Kentucky**	67	72	79	52	64	94	80	96	98	83	83	94
**Louisiana**	64	49	63	40	44	58	57	69	71	59	61	63
**Maine**	58	83	88	66	56	83	83	99	100	89	93	87
**Maryland**	53	58	78	52	55	85	82	82	85	74	77	91
**Massachusetts**	57	55	69	45	54	73	67	77	79	68	71	80
**Michigan**	44	59	72	49	53	73	68	79	76	68	68	77
**Minnesota**	51	62	74	47	52	75	78	78	81	69	72	78
**Mississippi**	54	83	68	41	45	65	64	84	92	75	79	99
**Missouri**	56	59	71	46	52	75	73	80	80	68	69	81
**Montana**	49	62	81	56	47	81	67	95	89	81	77	81
**Nebraska**	78	73	79	49	55	87	80	87	98	79	85	86
**Nevada**	39	42	51	35	44	64	59	60	65	65	61	68
**New Hampshire**	60	49	78	41	50	69	68	79	79	67	67	82
**New Jersey**	51	56	66	41	47	67	63	76	78	69	71	75
**New Mexico**	75	69	64	47	61	79	95	76	86	77	74	85
**New York**	49	57	65	46	49	69	64	70	77	66	65	72
**North Carolina**	55	63	79	51	62	85	82	91	95	82	80	82
**North Dakota**	91	91	94	54	69	99	79	86	85	84	73	74
**Ohio**	48	54	68	44	46	68	68	73	78	66	67	72
**Oklahoma**	46	64	63	46	48	62	71	77	87	72	71	74
**Oregon**	48	50	68	43	50	71	69	74	79	46	34	38
**Pennsylvania**	52	61	74	48	55	75	75	88	91	78	77	84
**Rhode Island**	64	49	80	52	61	72	84	94	93	85	84	89
**South Carolina**	61	54	73	46	51	82	70	77	89	80	78	79
**South Dakota**	100	97	89	57	64	73	71	88	96	70	80	98
**Tennessee**	48	58	70	45	48	76	76	87	96	78	81	88
**Texas**	45	52	64	41	47	64	60	70	73	63	66	70
**Utah**	43	52	66	37	42	53	59	70	69	65	66	67
**Vermont**	63	95	74	67	44	70	55	93	85	91	76	92
**Virginia**	40	48	63	40	35	37	33	76	82	67	55	53
**Washington**	46	52	65	46	51	70	66	78	77	70	68	74
**West Virginia**	86	81	67	75	69	95	100	100	98	94	81	93
**Wisconsin**	57	61	71	43	50	67	71	74	73	64	63	68
**Wyoming**	85	100	88	50	55	52	84	91	86	90	78	80

Table A3. Smoothing Parameters for the Holt-Winters' Forecasting by State

State	alpha (α)	beta (β*)	gamma (γ)
**Alabama**	0.1050	0.0162	0.7013
**Alaska**	0.0491	0.0877	0.4980
**Arizona**	0.1491	0.0381	0.4310
**Arkansas**	0.0426	0.0882	0.6637
**California**	0.2817	0.0000	0.6552
**Colorado**	0.0685	0.0000	0.3896
**Connecticut**	0.1059	0.0000	0.4359
**Delaware**	0.1665	0.0208	0.4038
**District of Columbia**	0.0923	0.0000	0.5157
**Florida**	0.1447	0.0195	0.6686
**Georgia**	0.2049	0.0321	0.4613
**Hawaii**	0.0146	0.6730	0.6628
**Idaho**	0.0090	1.0000	0.5640
**Illinois**	0.1829	0.0274	0.4946
**Indiana**	0.0525	0.0334	0.5058
**Iowa**	0.1160	0.0540	0.5406
**Kansas**	0.0701	0.0122	0.4871
**Kentucky**	0.0604	0.0494	0.4764
**Louisiana**	0.0893	0.1310	0.4797
**Maine**	0.0274	0.1580	0.3866
**Maryland**	0.0263	0.0000	0.4569
**Massachusetts**	0.0841	0.0554	0.5767
**Michigan**	0.1009	0.0239	0.6195
**Minnesota**	0.1291	0.0088	0.5211
**Mississippi**	0.1009	0.0000	0.6027
**Missouri**	0.0972	0.0000	0.5170
**Montana**	0.0954	0.0783	0.3634
**Nebraska**	0.0566	0.1721	0.2803
**Nevada**	0.0321	0.0490	0.6138
**New Hampshire**	0.0902	0.0775	0.4581
**New Jersey**	0.1657	0.0260	0.4642
**New Mexico**	0.1470	0.0072	0.3644
**New York**	0.1818	0.0245	0.5085
**North Carolina**	0.1209	0.0098	0.4294
**North Dakota**	0.0459	0.0485	0.4518
**Ohio**	0.0997	0.1281	0.3487
**Oklahoma**	0.2460	0.0389	0.5948
**Oregon**	0.1868	0.0000	0.5835
**Pennsylvania**	0.1784	0.0215	0.5000
**Rhode Island**	0.1205	0.0124	0.5135
**South Carolina**	0.0805	0.0851	0.2857
**South Dakota**	0.1044	0.0957	0.4531
**Tennessee**	0.0779	0.0372	0.5314
**Texas**	0.1822	0.0000	0.4703
**Utah**	0.0448	0.0430	0.4546
**Vermont**	0.0945	0.0330	0.4606
**Virginia**	0.5253	0.0000	0.8483
**Washington**	0.1281	0.0209	0.4646
**West Virginia**	0.0755	0.0453	0.4634
**Wisconsin**	0.1103	0.0241	0.6439
**Wyoming**	0.0826	0.1182	0.4289

Table A4. Coefficients for the Holt-Winters' Forecastings by State

	a	b	s1	s2	s3	s4	s5	s6	s7	s8	s9	s10	s11	s12
**AL**	27.42	-0.03	6.89	8.01	7.80	8.62	3.85	5.88	-0.34	2.91	5.36	12.26	2.44	4.88
**AK**	40.03	0.25	18.27	5.19	1.36	12.3	22.14	6.02	5.59	5.90	8.89	8.78	13.97	3.01
**AZ**	69.21	0.15	10.48	19.51	9.27	14.7	0.40	-3.72	-10.33	-0.01	-2.12	8.94	5.75	6.45
**AR**	32.11	0.08	11.38	10.28	8.23	11.0	2.22	-1.70	2.75	-1.77	14.8	7.41	8.68	1.56
**CA**	67.85	0.11	4.71	1.43	4.29	4.19	2.15	-10.63	-13.96	-12.67	-5.53	-1.71	-0.40	-3.70
**CO**	54.69	-0.08	-2.42	4.61	8.37	9.81	4.61	-0.35	-1.59	1.27	3.21	5.99	0.83	-2.45
**CT**	58.75	0.01	-2.08	0.88	0.08	7.65	0.51	-3.06	-8.06	-7.41	3.88	1.24	-1.54	-4.23
**DC**	58.46	-0.09	-2.35	-3.71	0.45	4.03	7.67	-6.60	-6.42	-3.51	1.25	2.11	-4.05	2.25
**DE**	21.62	-0.15	3.10	6.64	7.49	4.81	6.08	3.14	4.57	3.89	7.41	9.57	4.92	8.63
**FL**	70.72	0.06	-3.60	-0.12	-0.05	-2.19	-6.04	-8.45	-8.75	-10.65	-4.73	-0.48	-1.97	-7.95
**GA**	71.98	0.10	-5.87	-3.70	6.21	11.0	-4.32	-10.44	-9.74	-6.82	-1.93	-0.18	-1.72	-5.00
**HI**	15.87	0.06	4.97	9.97	10.18	9.70	2.19	6.44	2.52	0.43	8.84	7.21	2.48	5.21
**ID**	28.41	0.01	2.77	1.29	5.85	5.20	3.58	2.32	3.66	6.95	8.46	9.85	4.85	2.67
**IL**	55.00	0.00	0.57	1.09	2.85	7.97	4.34	-2.93	-2.40	-2.49	4.16	10.01	5.10	-1.56
**IN**	60.80	-0.02	-2.92	-5.85	0.56	6.84	-1.90	-9.62	-13.63	-6.00	-0.92	2.18	-1.71	-6.39
**IA**	35.73	0.06	-8.69	-6.07	1.02	-1.72	-2.74	-8.36	-8.67	-7.87	-5.42	-0.24	-6.21	-6.19
**KS**	29.79	-0.13	2.03	5.58	6.96	7.19	4.50	4.38	-0.43	6.63	6.57	10.74	0.60	-7.72
**KY**	39.66	0.04	5.51	3.14	4.30	5.31	6.17	2.29	4.65	2.76	9.29	6.25	6.65	0.89
**LA**	38.63	-0.05	8.32	9.87	11.57	6.96	2.77	0.58	-4.60	3.40	1.11	10.80	8.72	-0.06
**ME**	52.14	0.12	7.53	5.14	17.53	11.5	5.48	1.57	-5.89	-1.72	9.17	12.08	13.91	7.92
**MD**	64.07	0.01	-4.38	-2.99	-1.80	7.94	1.47	-10.15	-5.29	-8.14	2.58	5.83	2.85	-3.81
**MA**	45.67	0.05	2.61	3.99	5.96	6.61	8.90	-1.26	-3.03	-1.90	7.41	6.26	6.35	3.95
**MI**	64.56	0.04	-8.75	-3.33	0.59	2.26	-1.66	-6.55	-13.19	-11.45	-2.61	-0.14	-4.01	-4.58
**MN**	56.21	-0.19	4.86	8.46	4.26	11.8	8.08	-1.28	-3.49	-1.41	7.16	11.42	7.37	5.21
**MS**	43.92	-0.22	-0.27	2.54	11.47	14.9	3.34	-5.71	0.86	-1.29	1.73	15.53	4.36	-0.29
**MO**	61.87	0.01	0.02	-1.09	-3.40	4.83	-0.57	-8.90	-9.70	-7.99	-1.50	2.06	-1.30	-3.40
**MT**	41.50	-0.04	0.46	7.20	10.72	4.97	6.30	6.63	2.03	-1.68	10.6	5.85	2.58	1.58
**NE**	41.78	0.18	-2.37	2.35	3.91	9.18	3.38	-1.05	2.72	3.74	3.91	6.07	-0.02	-2.02
**NV**	35.65	0.08	13.62	21.59	19.62	14.7	11.32	5.52	-2.01	2.55	3.11	5.61	8.26	2.12
**NH**	39.74	0.20	9.90	3.84	8.49	8.16	11.15	-5.53	0.35	-0.09	7.76	18.00	6.88	2.66
**NJ**	73.25	0.10	-3.38	-4.18	-1.22	7.08	3.99	-9.22	-10.01	-16.13	-4.15	5.06	-1.75	-3.62
**NM**	51.18	0.07	-0.53	1.35	1.93	1.52	-5.30	-6.87	-11.80	-8.40	-6.07	3.24	1.32	-9.66
**NY**	57.21	0.00	3.08	3.44	5.12	7.45	7.69	-2.12	-7.03	-9.90	0.23	7.17	6.13	4.04
**NC**	74.44	0.04	-5.21	-2.60	1.13	5.15	-5.03	-9.10	-9.61	-10.47	-2.49	0.94	0.77	-5.73
**ND**	8.29	-0.05	6.22	0.05	4.50	5.17	3.01	0.71	3.06	6.29	1.92	2.27	7.73	4.79
**OH**	50.09	0.05	-0.61	1.45	4.15	10.0	2.10	-2.71	-2.57	-2.66	2.57	6.42	3.27	-1.41
**OK**	47.43	0.13	7.67	9.41	7.08	9.96	2.45	3.14	3.14	7.37	12.1	11.51	7.66	2.27
**OR**	41.00	-0.23	-1.71	0.42	6.73	3.92	11.57	11.82	-0.56	-0.65	6.15	2.78	0.88	-3.18
**PA**	66.30	0.10	-4.80	-2.94	-1.10	4.99	1.12	-6.37	-8.97	-9.78	-0.76	5.71	-0.21	-6.30
**RI**	52.70	0.08	-14.61	-5.99	-2.57	4.28	-3.41	-13.80	-10.98	-16.87	-6.07	1.10	-9.84	-10.82
**SC**	41.38	-0.08	2.31	5.67	6.36	8.23	2.60	-3.13	-5.09	-4.33	1.37	5.68	3.29	0.66
**SD**	13.80	-0.03	2.91	3.66	9.35	7.16	4.73	4.23	1.56	2.18	2.26	3.29	3.51	-0.33
**TN**	65.42	0.08	-11.31	-6.03	-6.32	8.95	-7.26	-10.38	-13.09	-6.63	-0.82	-6.65	-6.78	-9.28
**TX**	73.10	-0.10	4.56	2.22	4.96	13.6	1.65	-9.83	-10.45	-6.94	2.25	6.22	4.11	-2.04
**UT**	56.92	0.05	-3.00	-5.14	3.93	-0.90	-4.96	-6.57	-11.50	-7.32	-3.27	1.85	-6.75	-8.76
**VT**	29.90	-0.04	12.23	10.60	0.83	11.5	6.20	0.49	-1.66	-3.09	5.08	11.63	20.28	5.04
**VA**	77.71	-0.32	-2.12	-0.68	5.15	12.8	7.04	-3.16	2.38	-1.00	7.60	3.32	-0.06	-0.78
**WA**	72.27	0.03	-3.31	0.71	4.07	5.81	2.07	-3.52	-8.45	-11.27	-5.87	-2.82	-6.01	-11.25
**WV**	35.61	0.08	3.74	8.13	14.29	13.0	5.31	4.06	5.54	2.53	7.07	11.44	9.41	6.47
**WI**	37.06	-0.09	3.57	6.21	2.00	13.5	3.93	0.47	0.40	-3.24	2.58	14.99	7.85	2.24
**WY**	13.31	0.07	9.10	5.16	7.87	6.86	3.00	3.92	3.04	6.00	1.38	8.21	5.36	4.46
