# Supplementary material for: Integrating Smart Health in the US Health Care System: Infodemiology Study of Asthma Monitoring in the Google Era
Source: JMIR Public Health Surveill. 2018 Mar 12;4(1):e24. doi: 10.2196/publichealth.8726 (PMC5869181; doi:10.2196/publichealth.8726)
Supplement: Multimedia Appendix 2 [file publichealth_v4i1e24_app2.pdf]

Multimedia Appendix 2: 'Asthma' Google Trends (2004-2015) vs. forecasts (2005-2020) by US State.

Figures B1 to B51 depict the changes in online interest in the term "asthma" from 2004 to 2015 and forecasts from 2016 to 2020 in each US State (and DC) in alphabetical order.

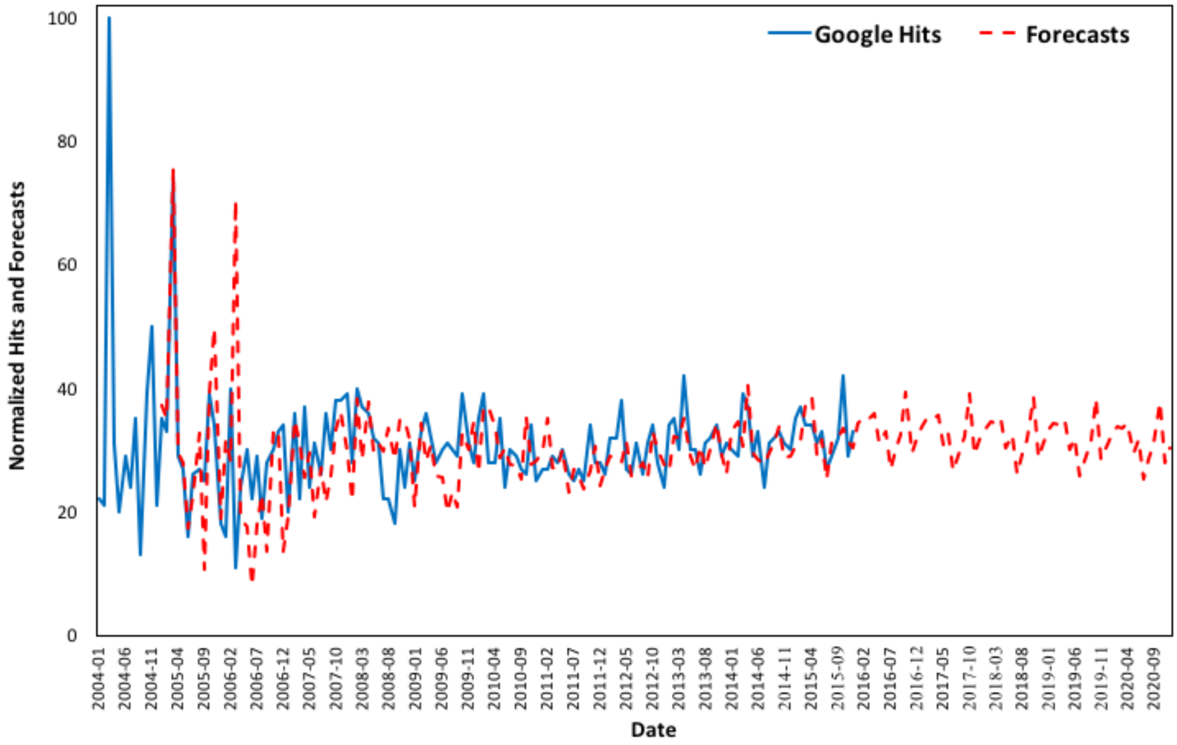

Figure B1. 'Asthma' Google Trends (2004-2015) vs. forecasts (2005-2020) in Alabama.

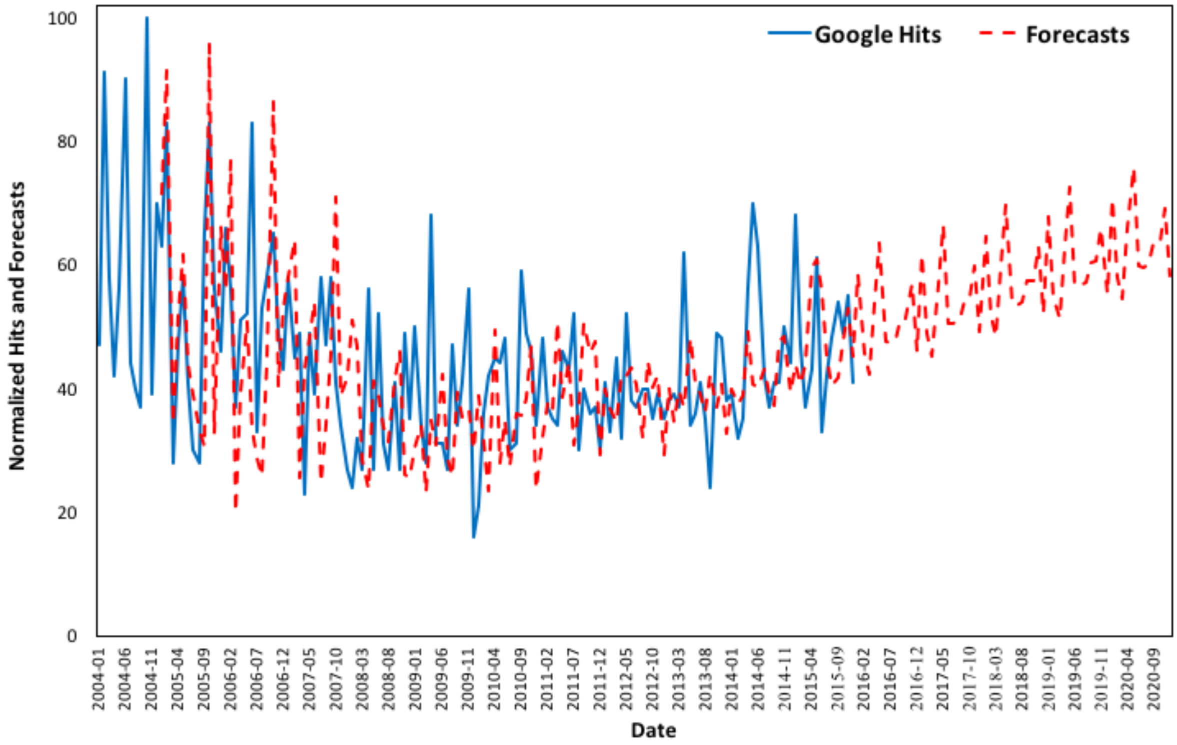

Figure B2. 'Asthma' Google Trends (2004-2015) vs. forecasts (2005-2020) in Alaska.

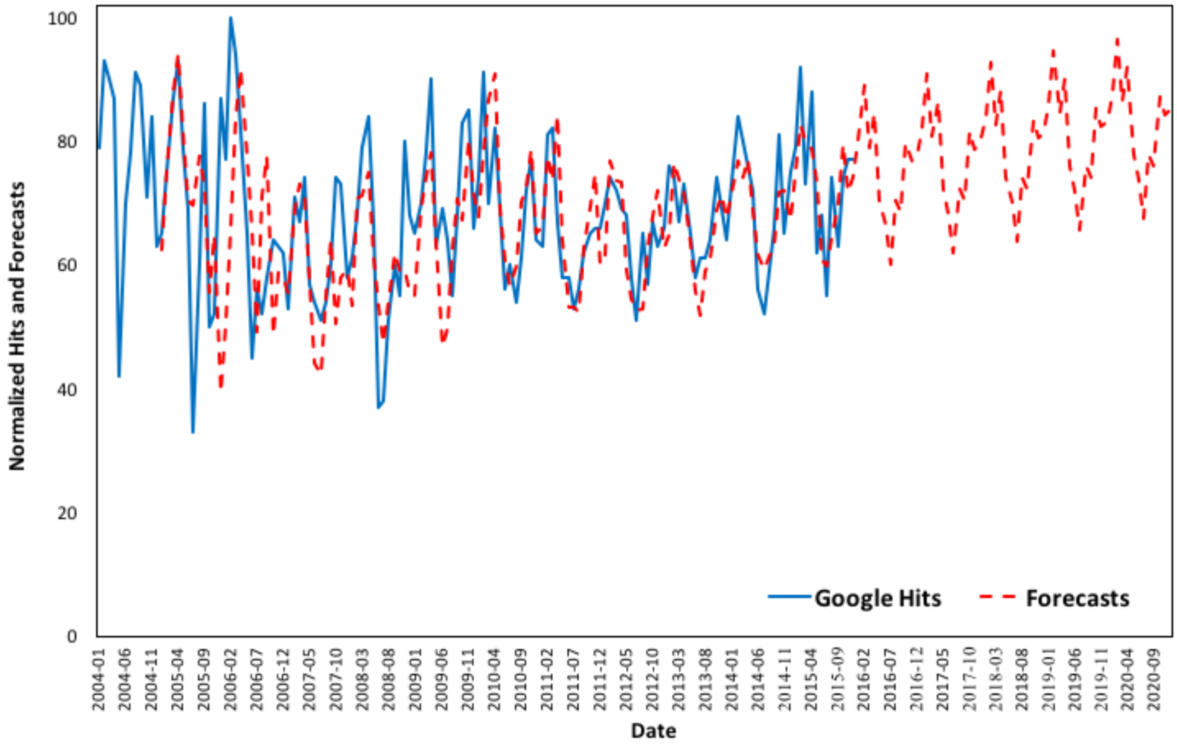

Figure B3. 'Asthma' Google Trends (2004-2015) vs. forecasts (2005-2020) in Arizona.

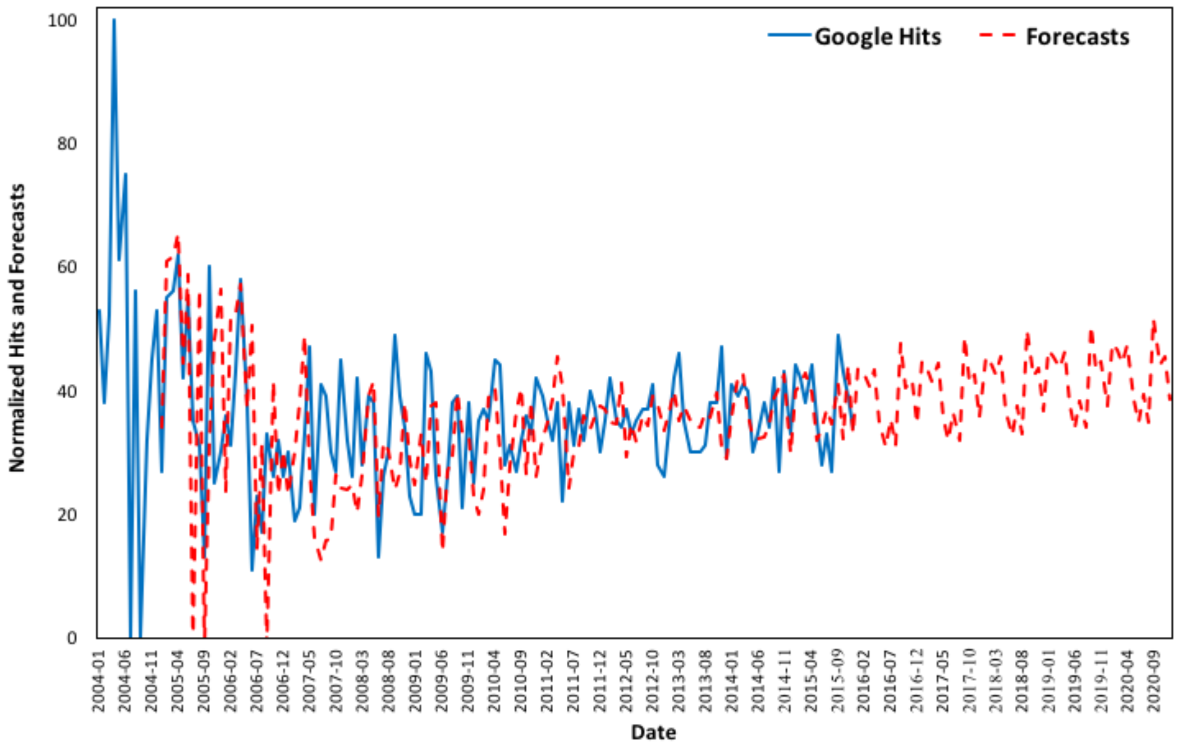

Figure B4. 'Asthma' Google Trends (2004-2015) vs. forecasts (2005-2020) in Arkansas.

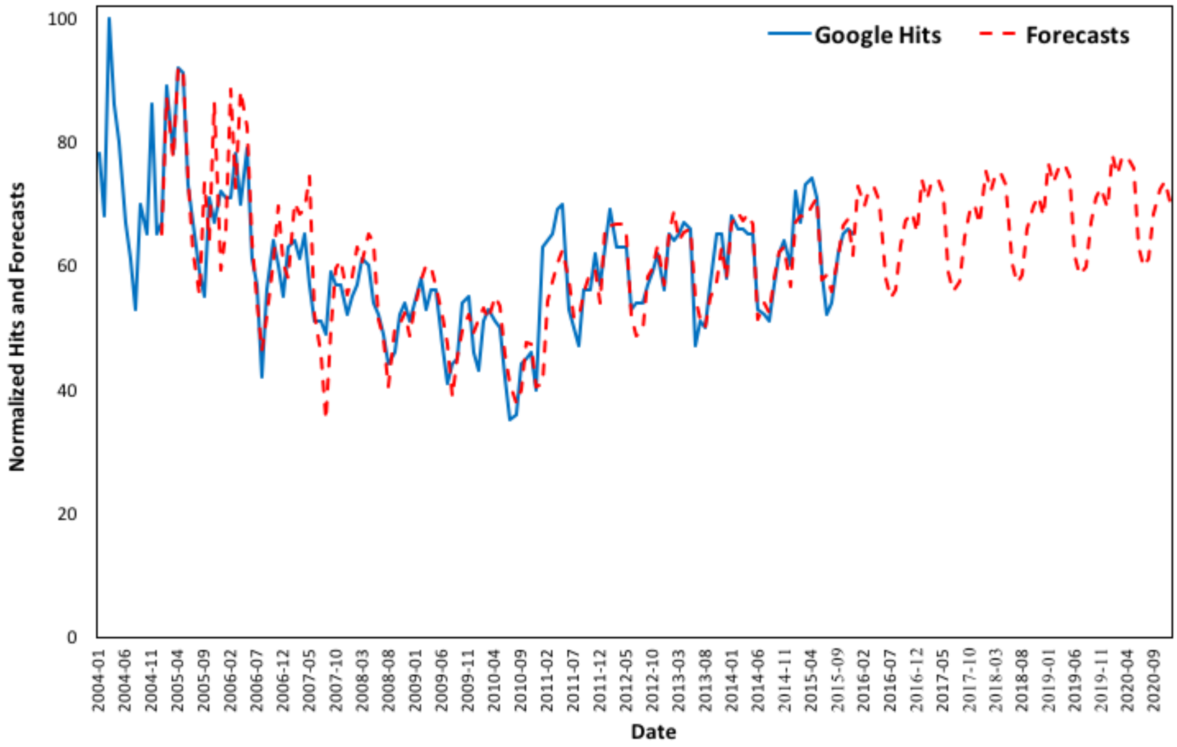

Figure B5. 'Asthma' Google Trends (2004-2015) vs. forecasts (2005-2020) in California.

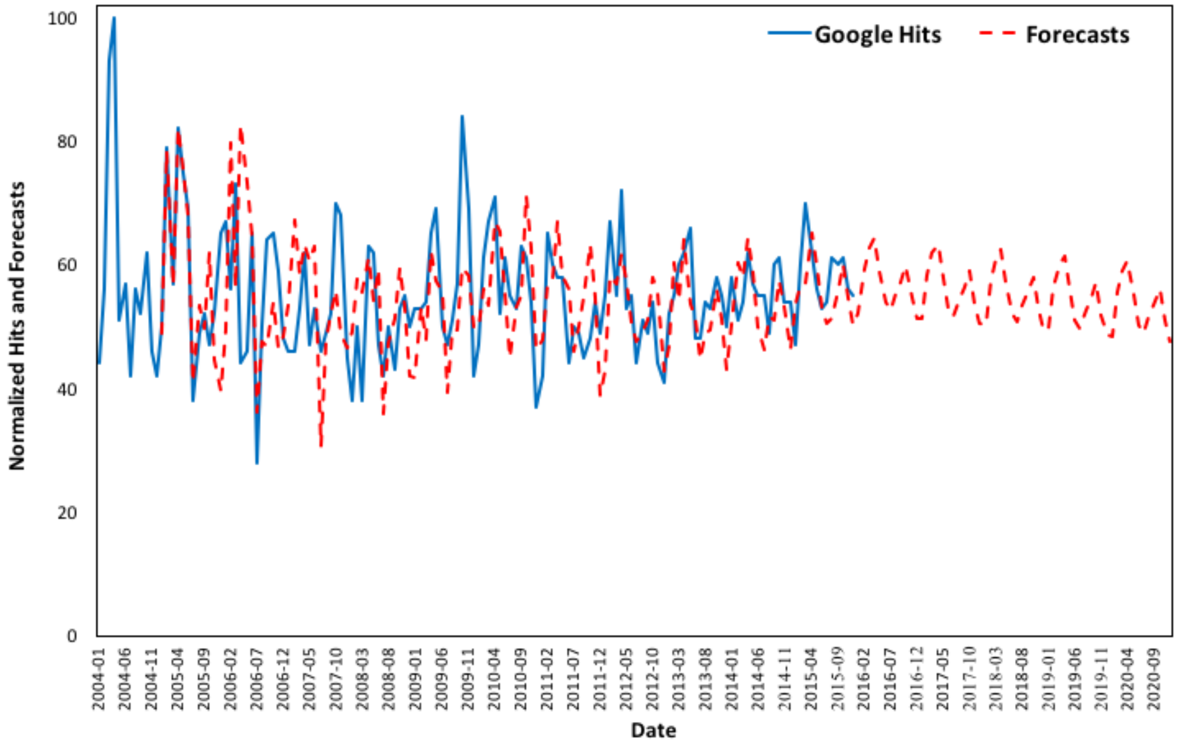

Figure B6. 'Asthma' Google Trends (2004-2015) vs. forecasts (2005-2020) in Colorado.

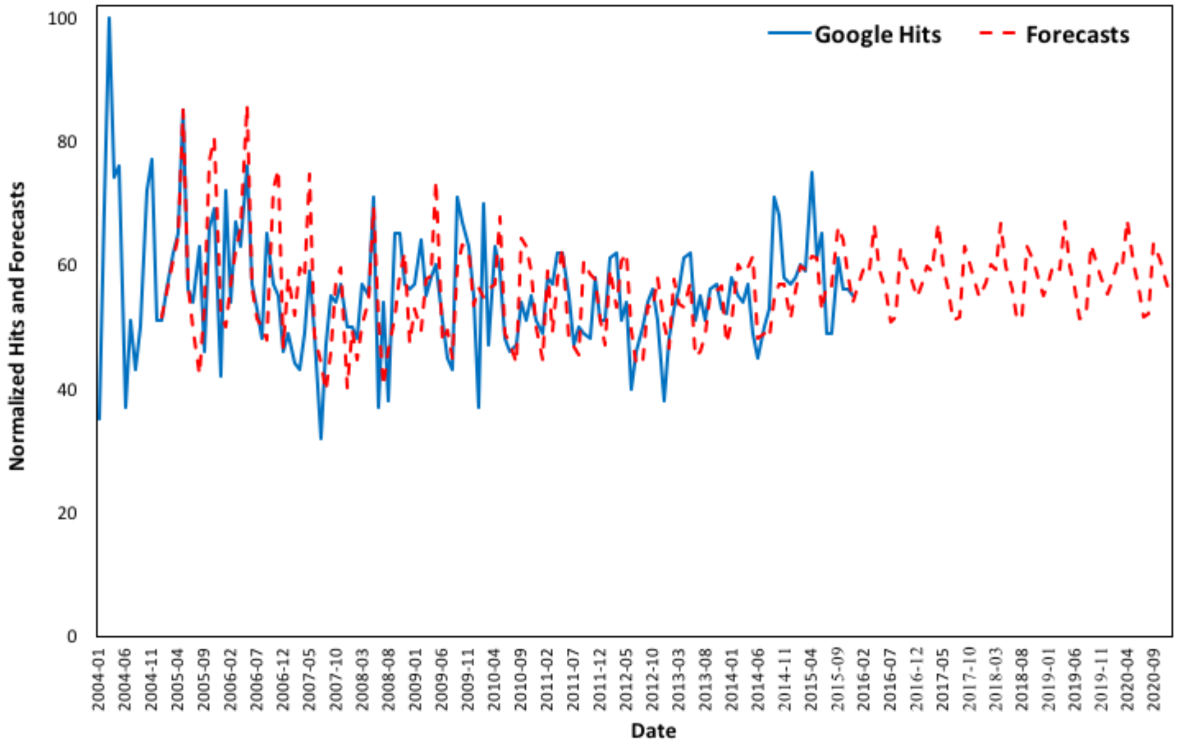

Figure B7. 'Asthma' Google Trends (2004-2015) vs. forecasts (2005-2020) in Connecticut.

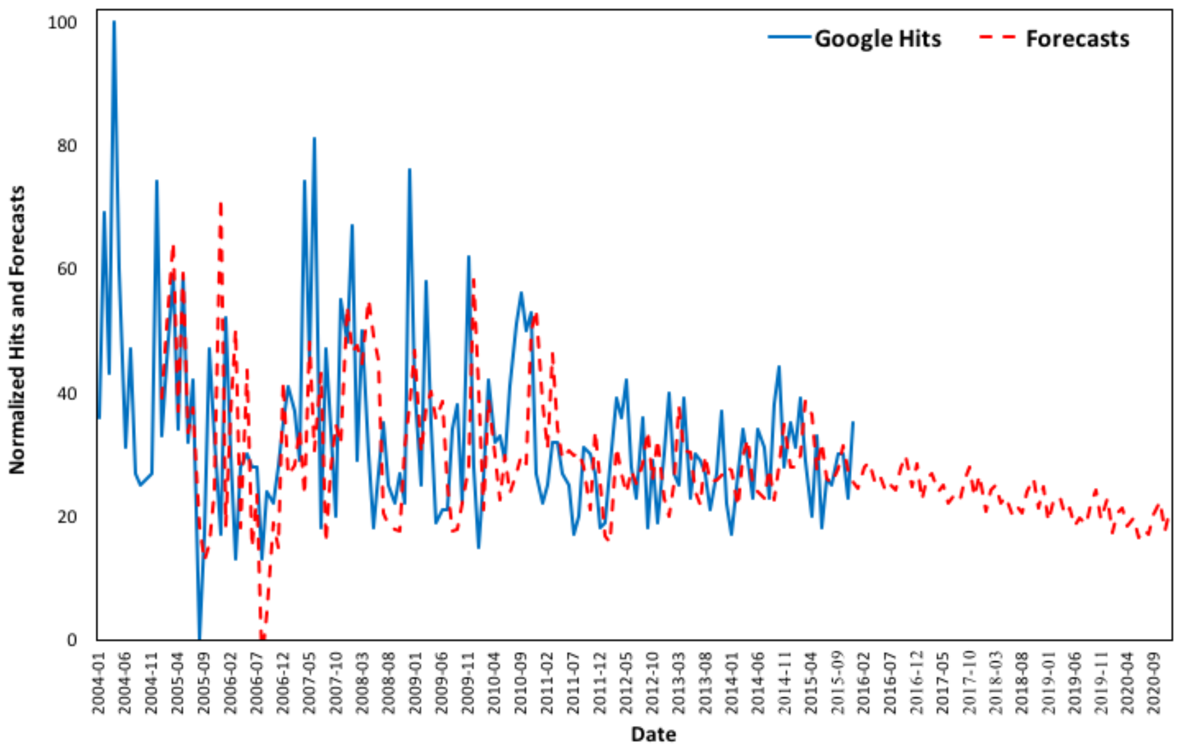

Figure B8. 'Asthma' Google Trends (2004-2015) vs. forecasts (2005-2020) in Delaware.

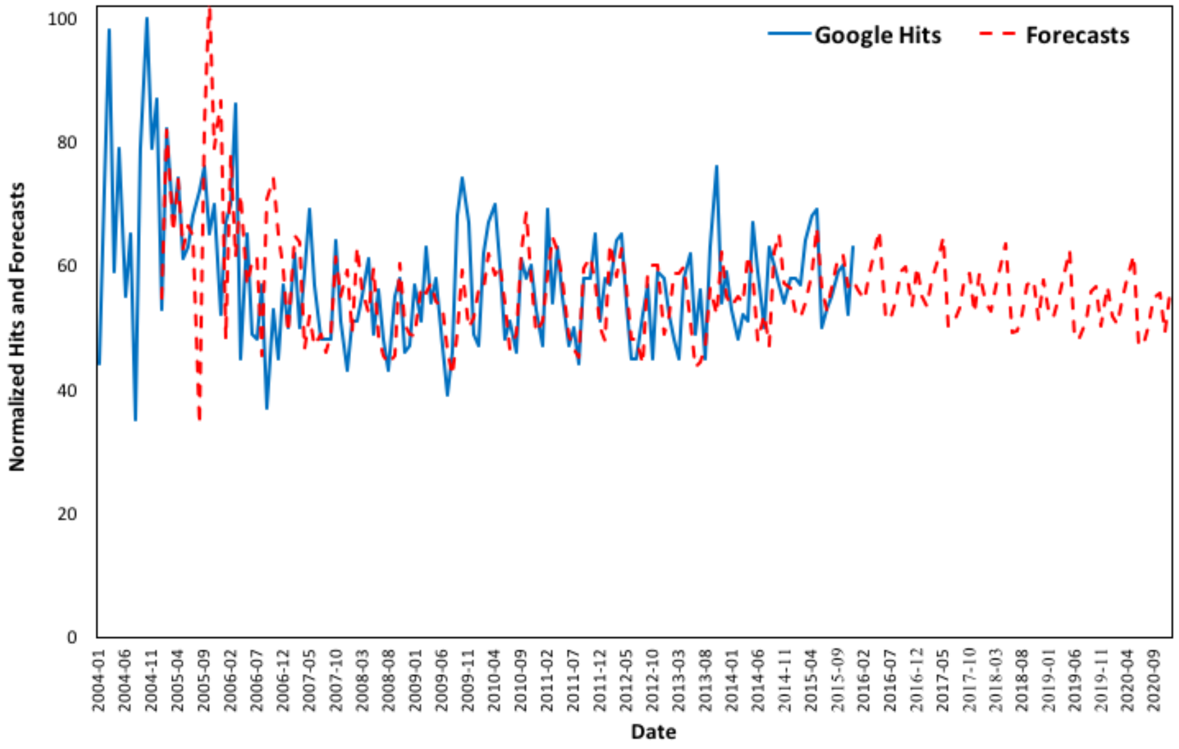

Figure B9. 'Asthma' Google Trends (2004-2015) vs. forecasts (2005-2020) in DC.

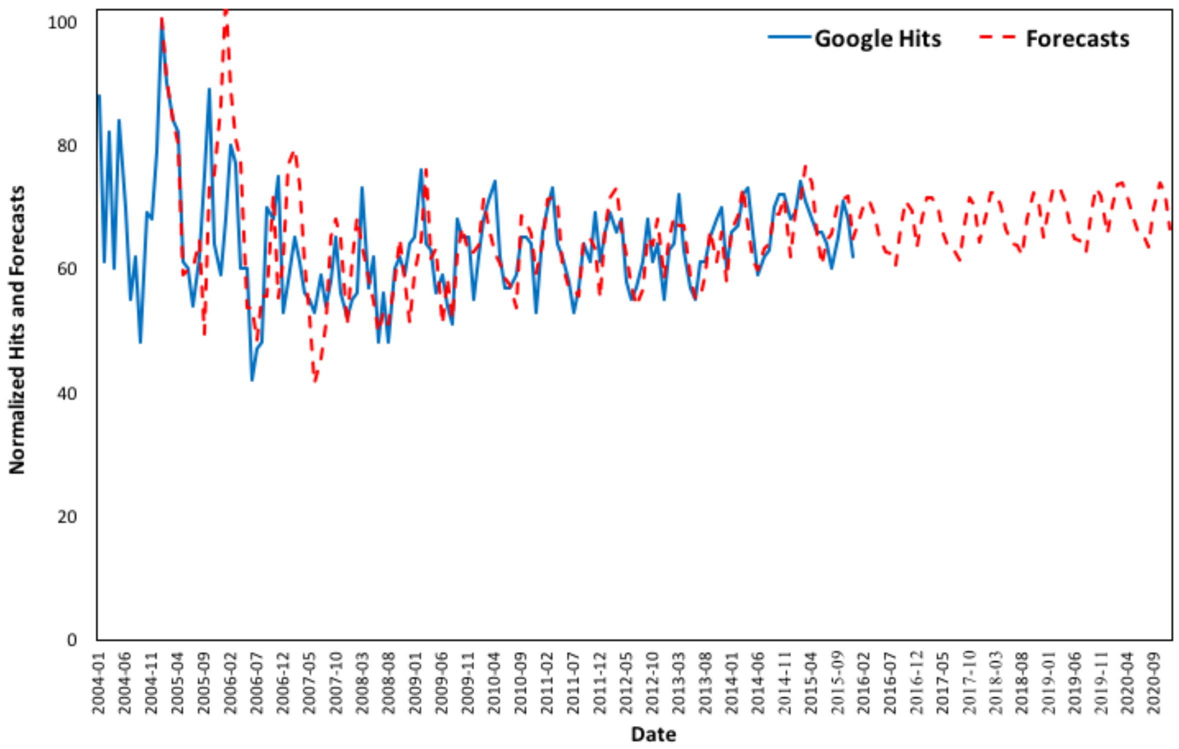

Figure B10. 'Asthma' Google Trends (2004-2015) vs. forecasts (2005-2020) in Florida.

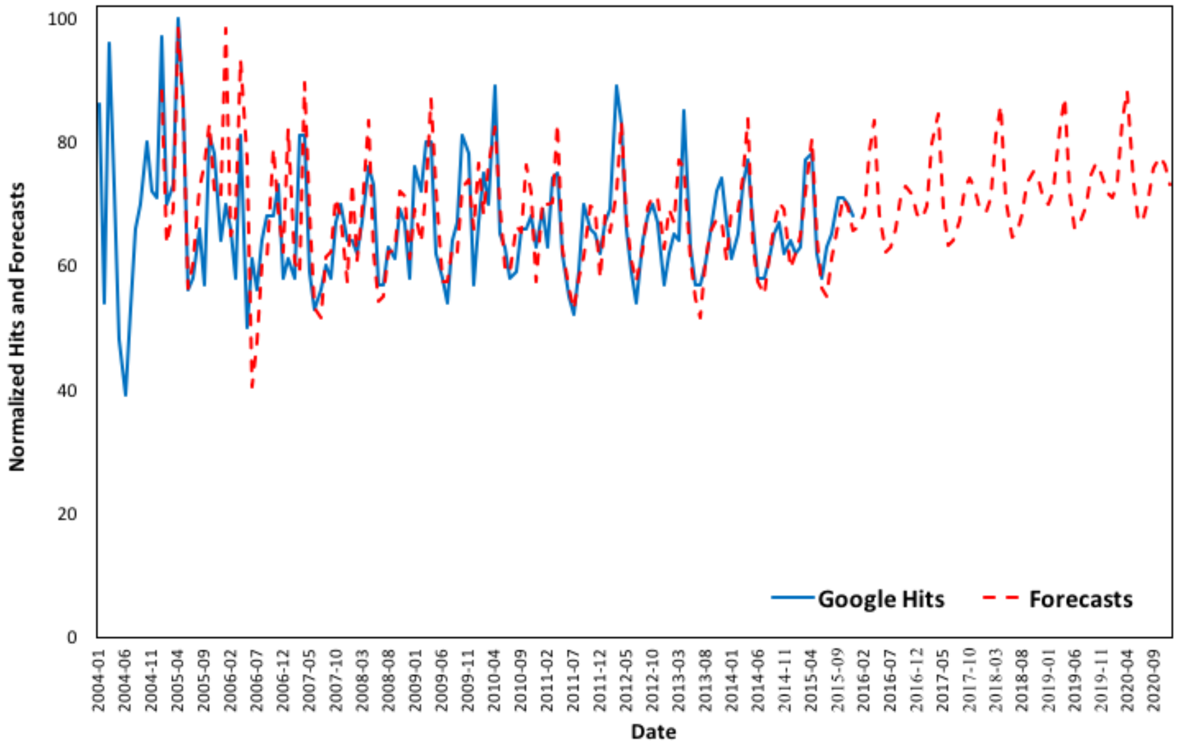

Figure B11. 'Asthma' Google Trends (2004-2015) vs. forecasts (2005-2020) in Georgia.

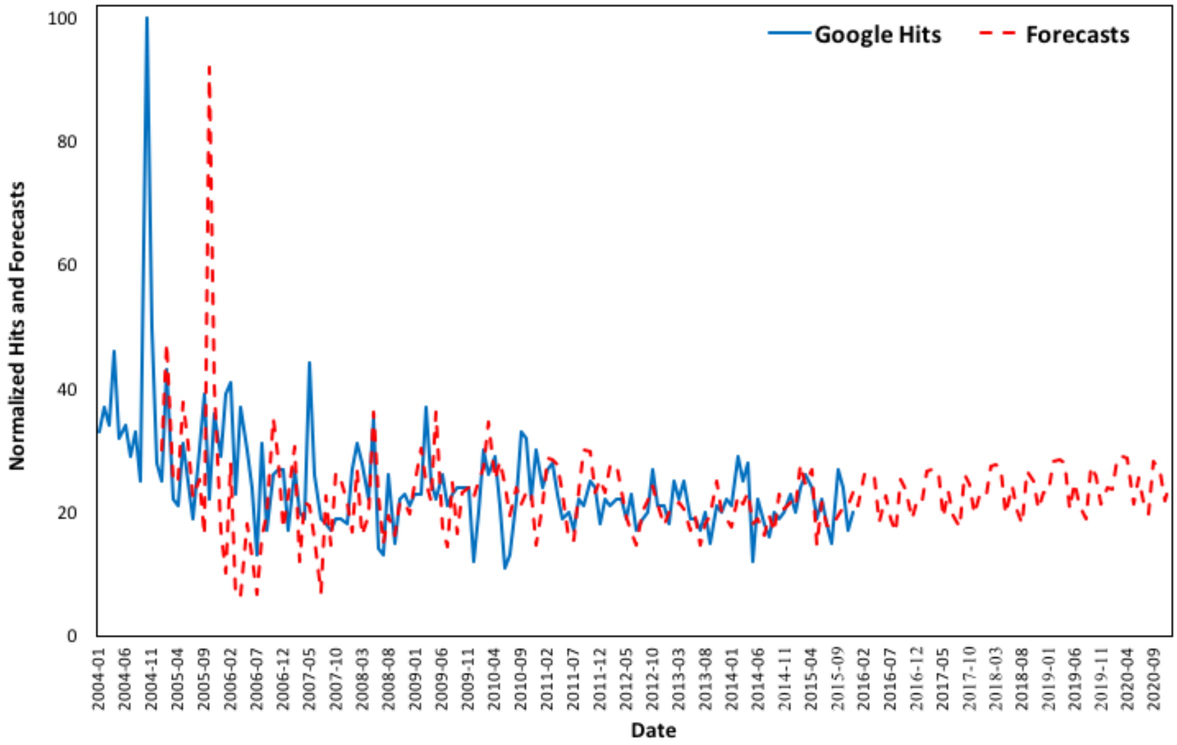

Figure B12. 'Asthma' Google Trends (2004-2015) vs. forecasts (2005-2020) in Hawaii.

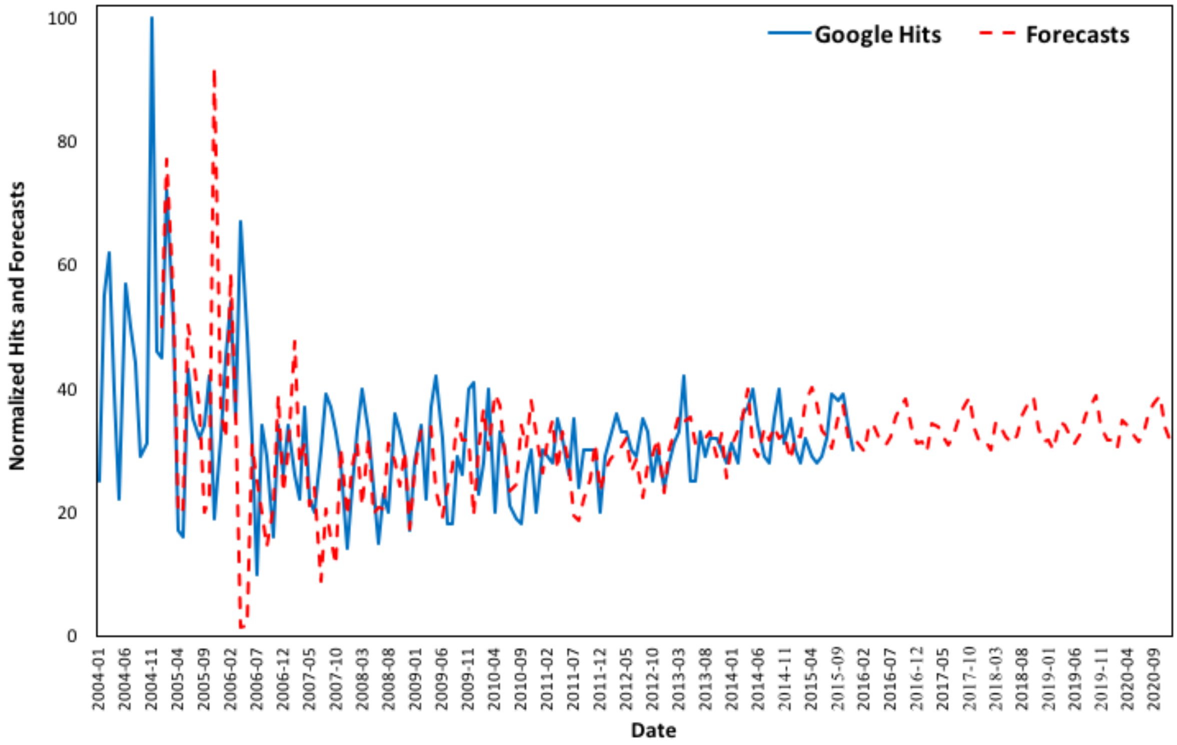

Figure B13. 'Asthma' Google Trends (2004-2015) vs. forecasts (2005-2020) in Idaho.

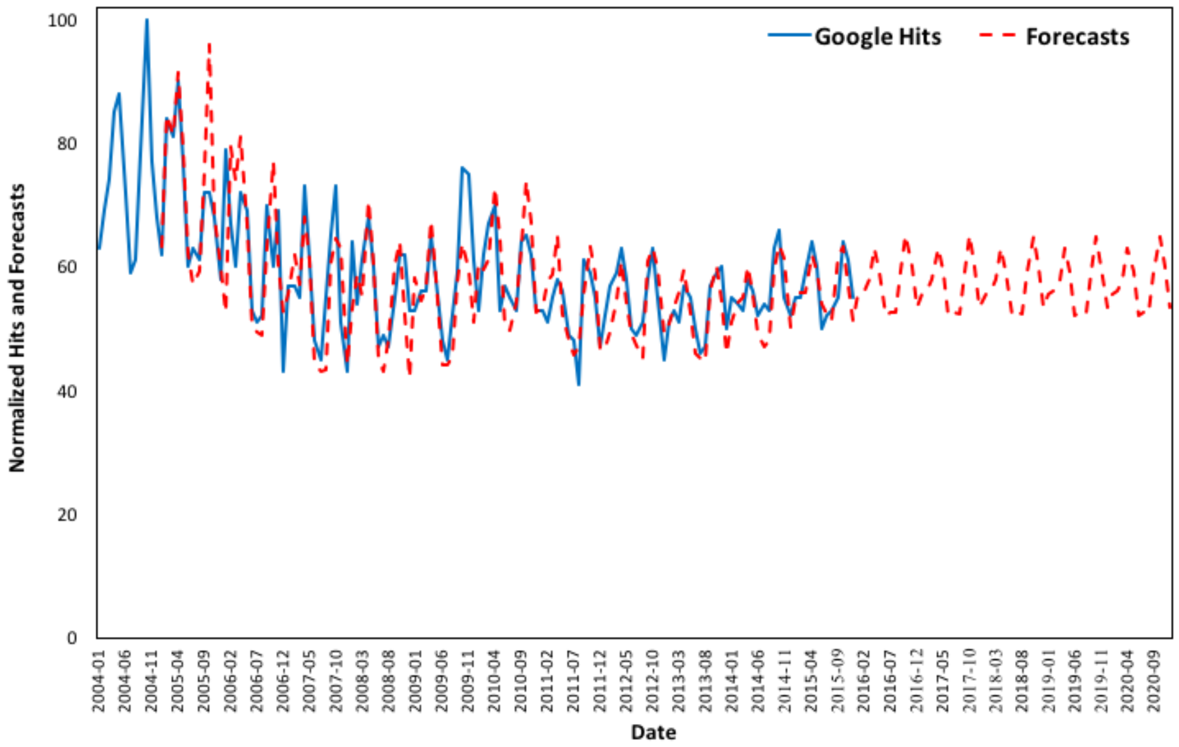

Figure B14. 'Asthma' Google Trends (2004-2015) vs. forecasts (2005-2020) in Illinois.

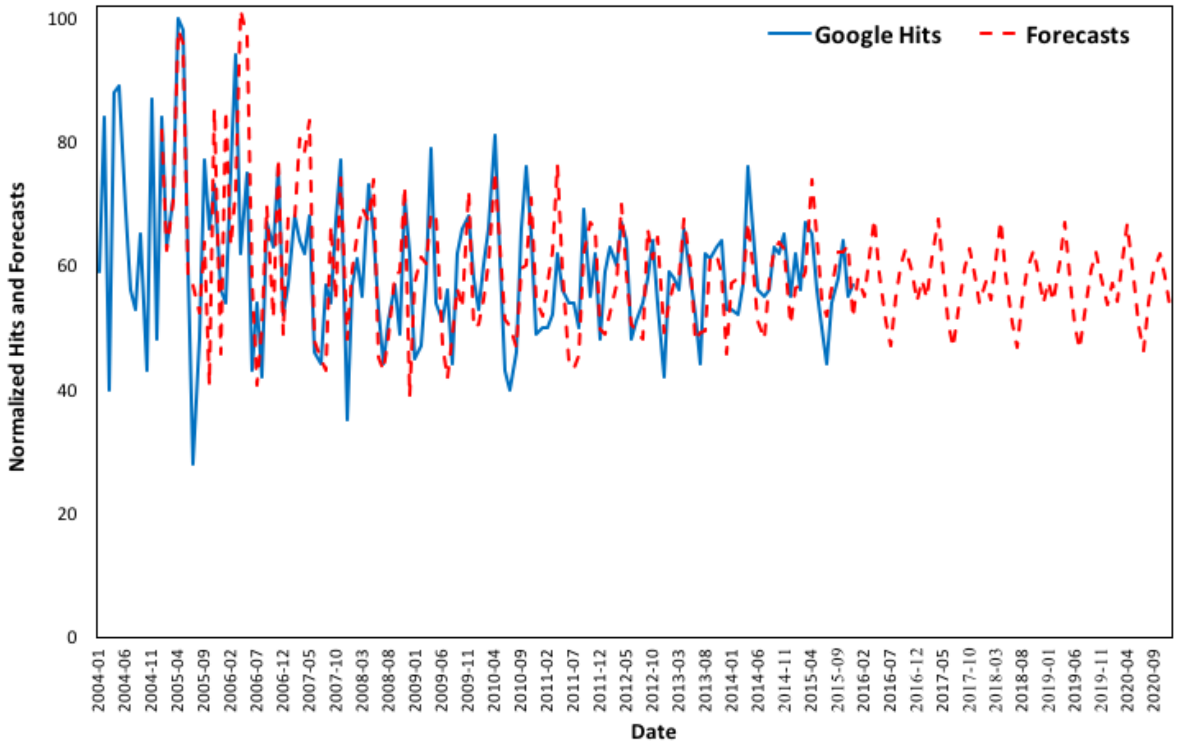

Figure B15. 'Asthma' Google Trends (2004-2015) vs. forecasts (2005-2020) in Indiana.

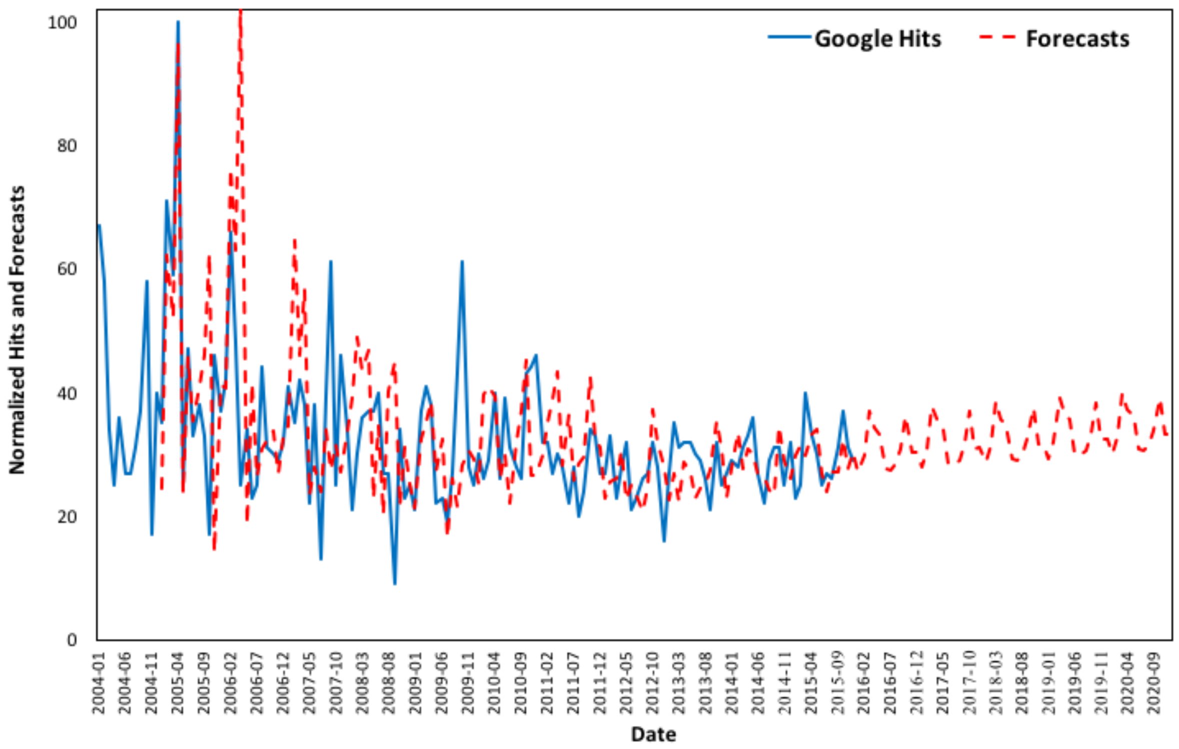

Figure B16. 'Asthma' Google Trends (2004-2015) vs. forecasts (2005-2020) in Iowa.

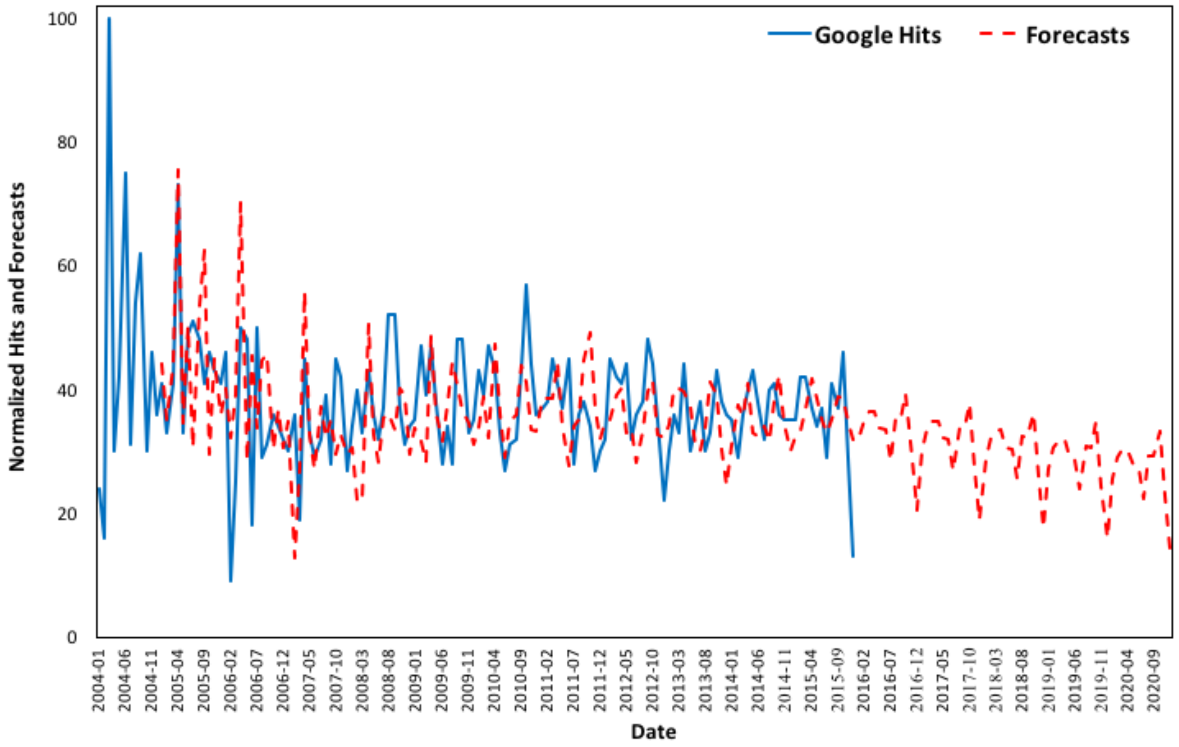

Figure B17. 'Asthma' Google Trends (2004-2015) vs. forecasts (2005-2020) in Kansas.

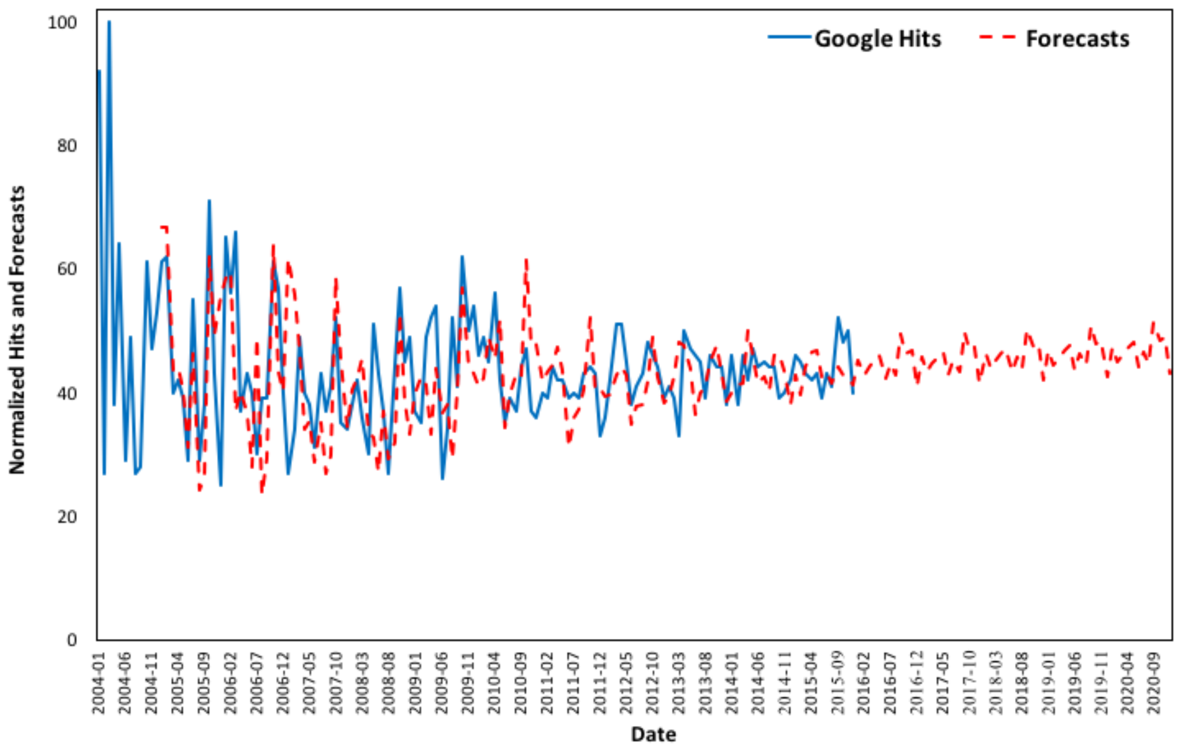

Figure B18. 'Asthma' Google Trends (2004-2015) vs. forecasts (2005-2020) in Kentucky.

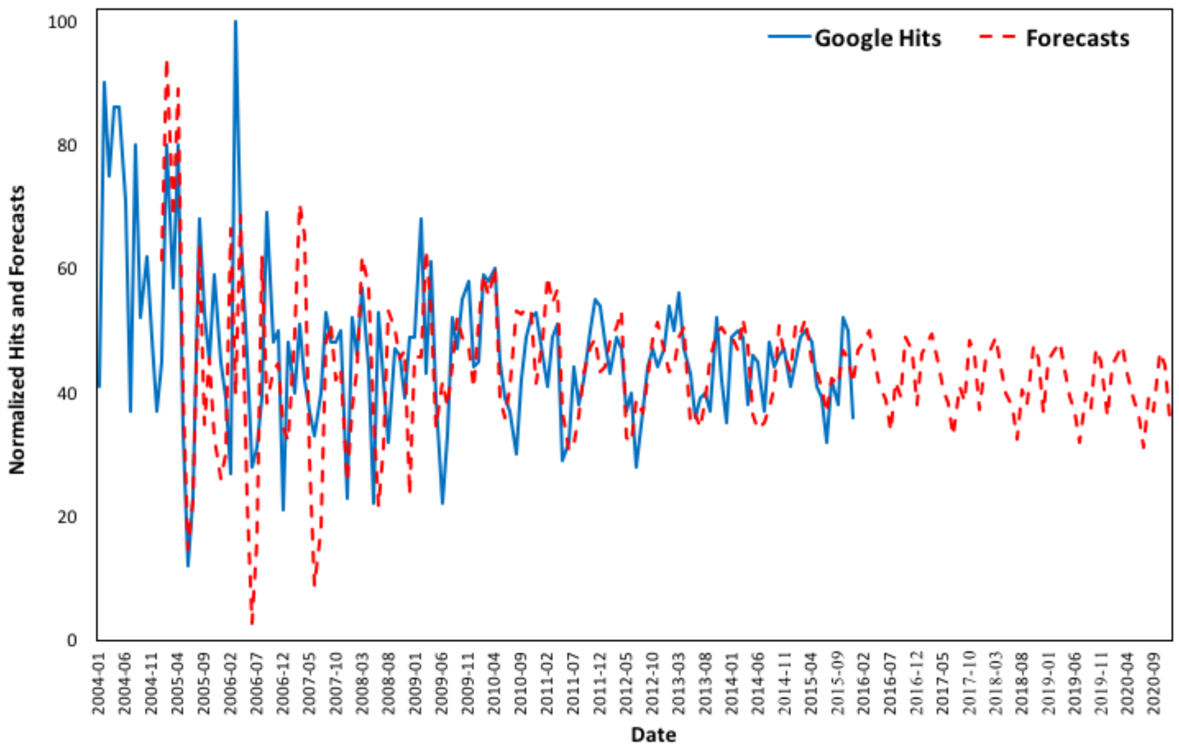

Figure B19. 'Asthma' Google Trends (2004-2015) vs. forecasts (2005-2020) in Louisiana.

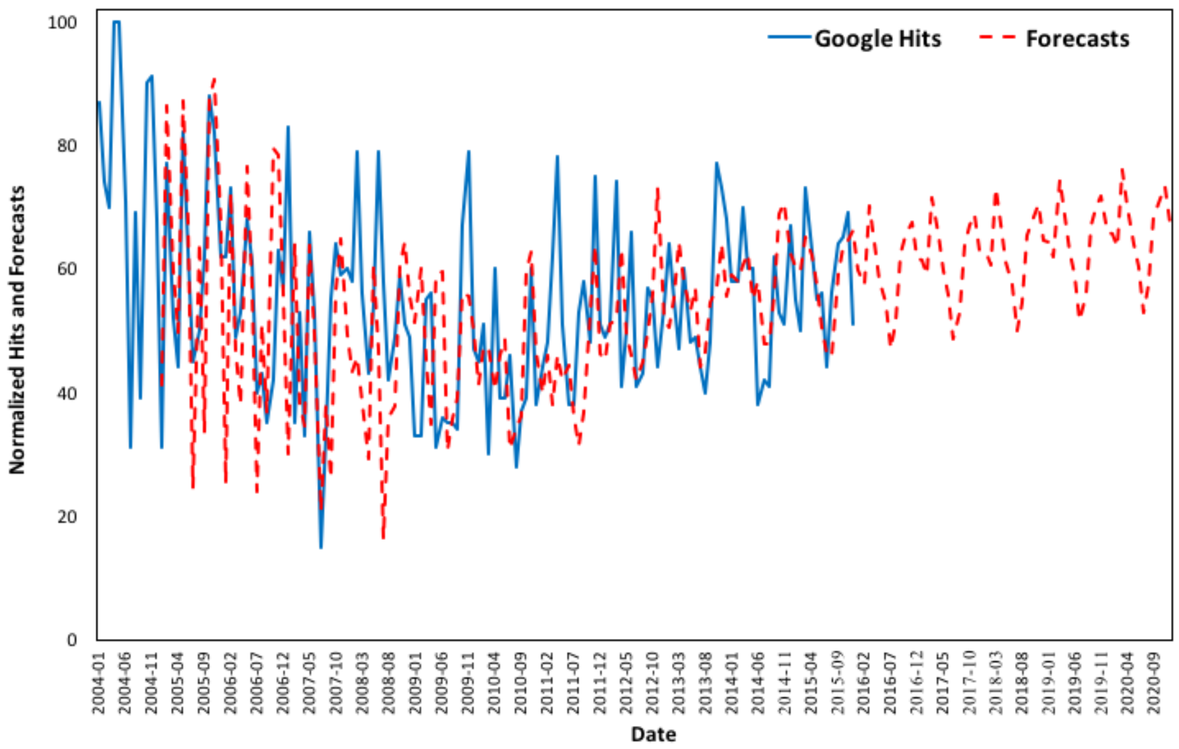

Figure B20. 'Asthma' Google Trends (2004-2015) vs. forecasts (2005-2020) in Maine.

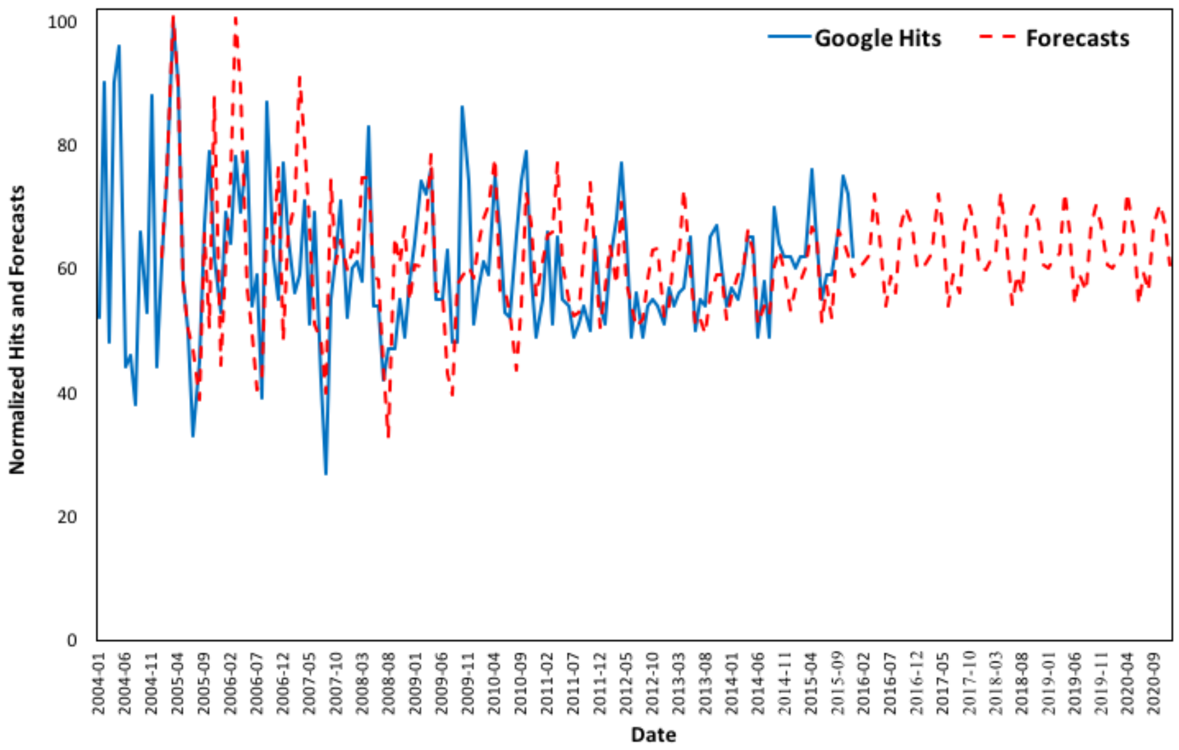

Figure B21. 'Asthma' Google Trends (2004-2015) vs. forecasts (2005-2020) in Maryland.

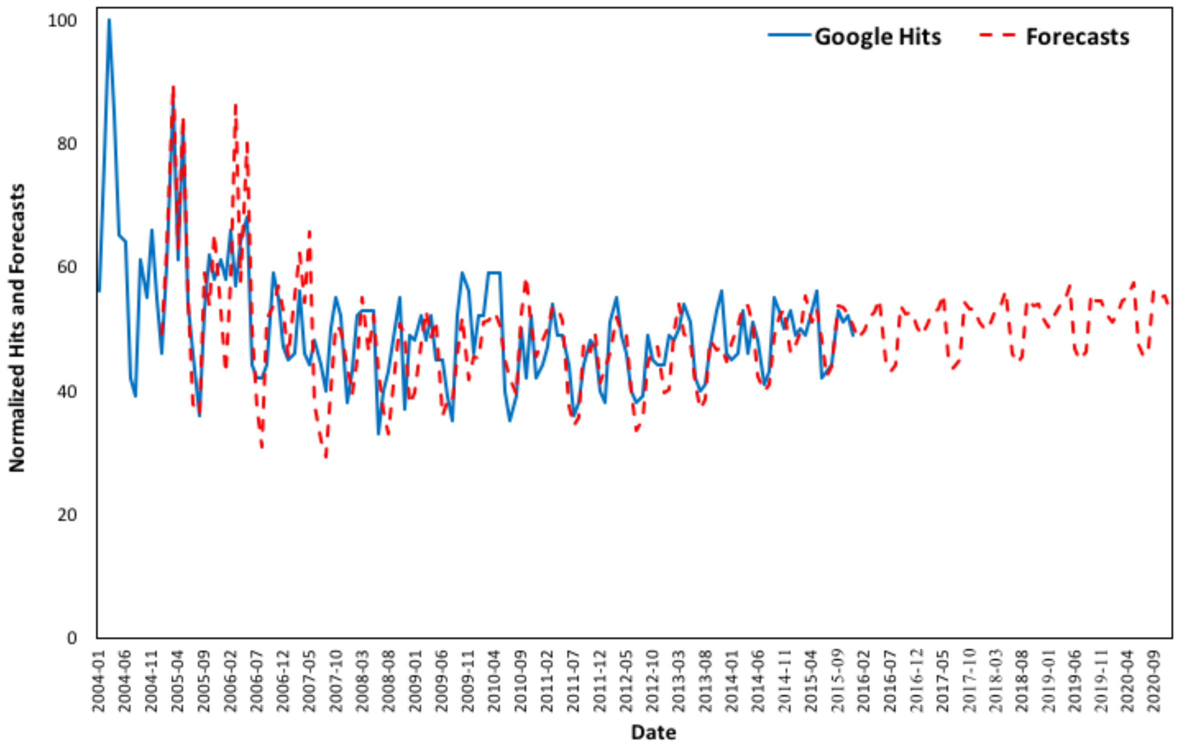

Figure B22. 'Asthma' Google Trends (2004-2015) vs. forecasts (2005-2020) in Massachusetts.

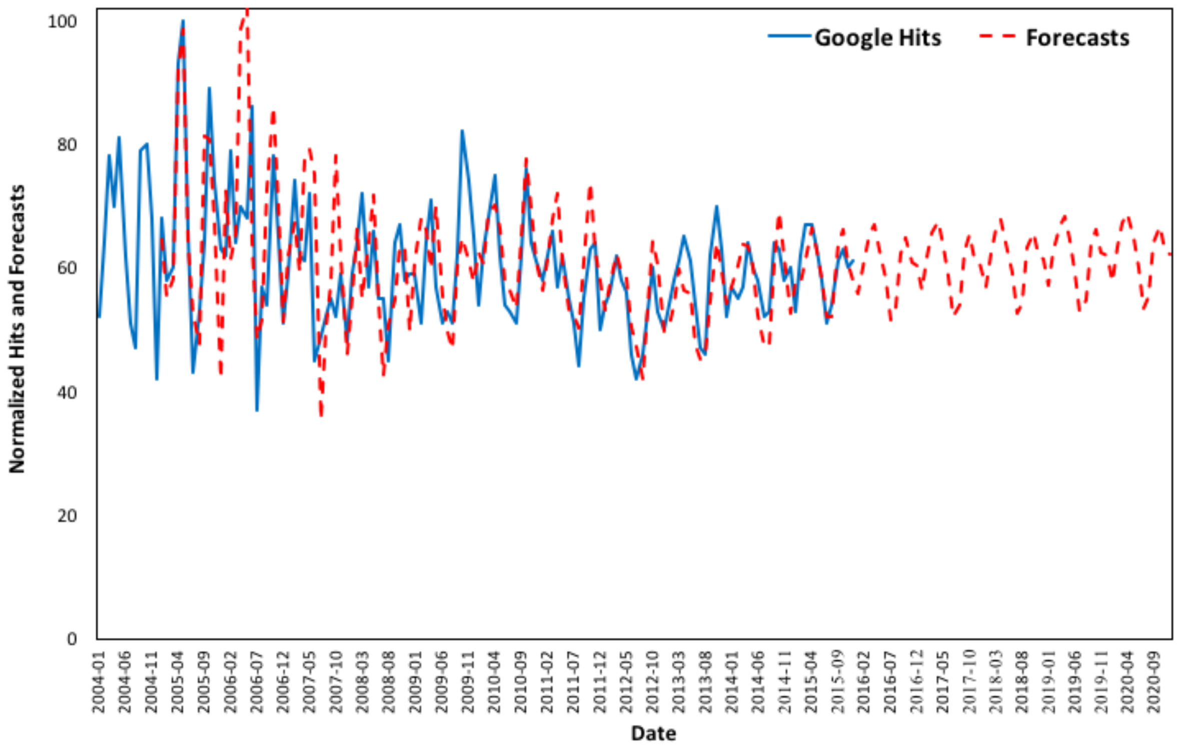

Figure B23. 'Asthma' Google Trends (2004-2015) vs. forecasts (2005-2020) in Michigan.

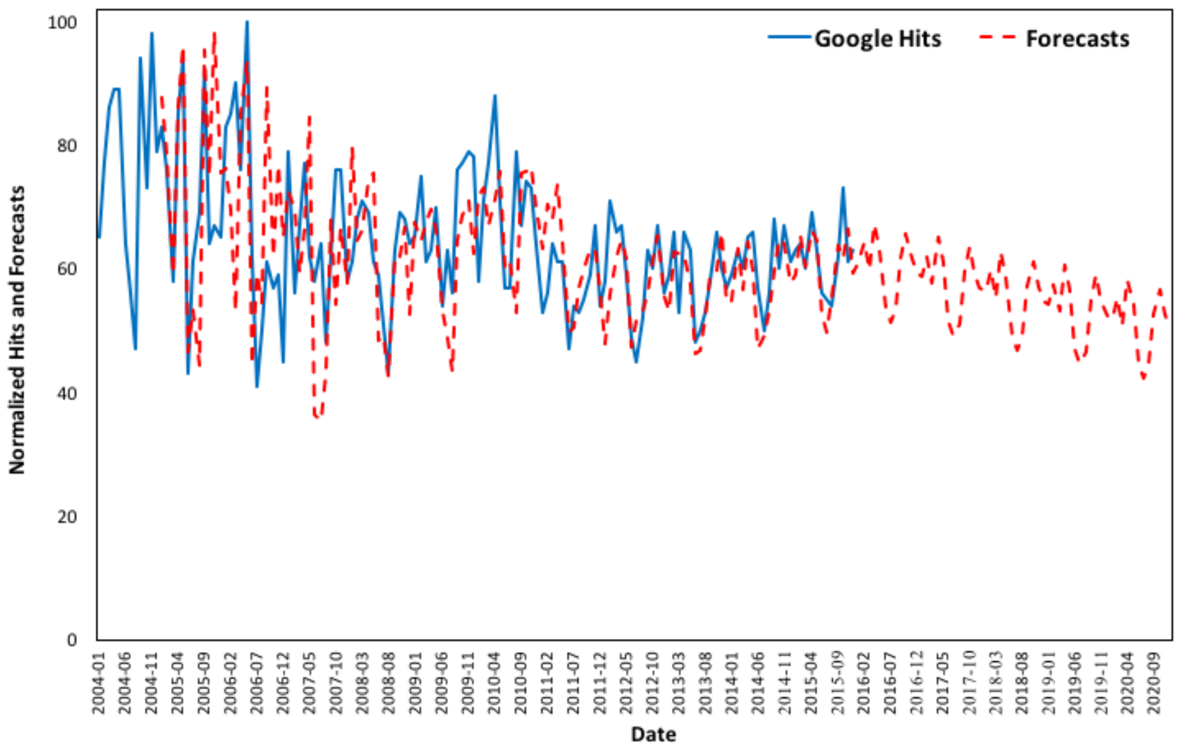

Figure B24. 'Asthma' Google Trends (2004-2015) vs. forecasts (2005-2020) in Minnesota.

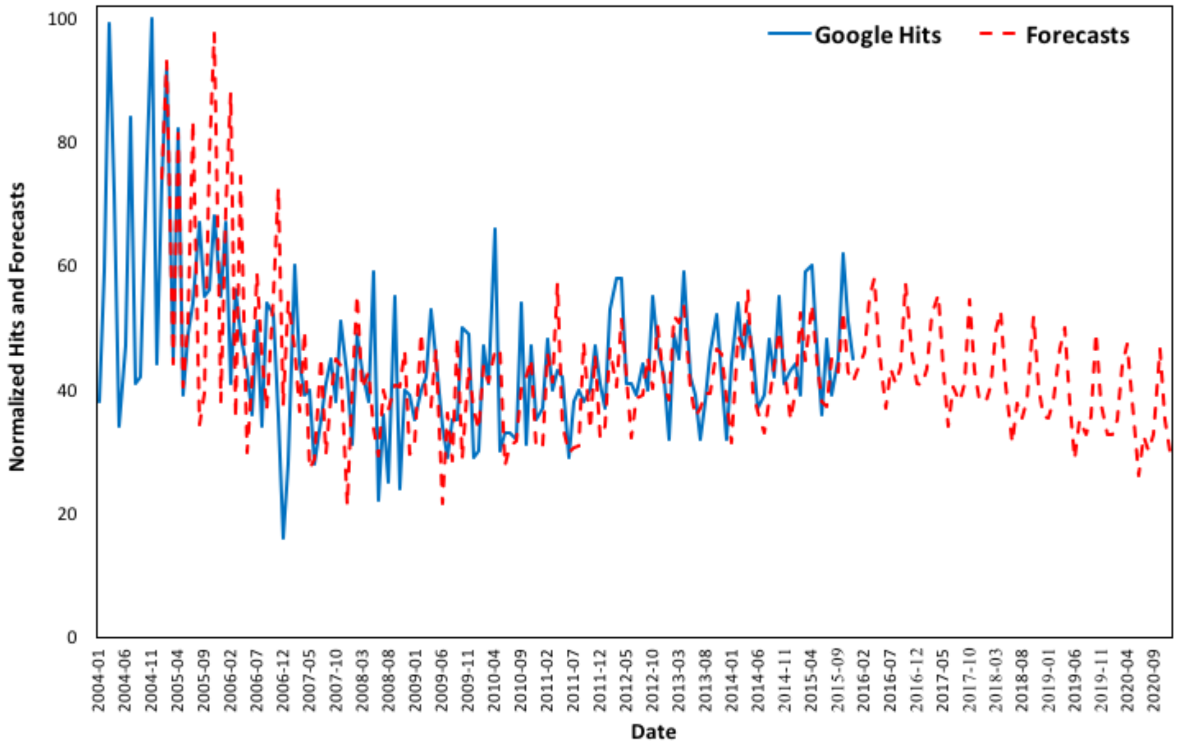

Figure B25. 'Asthma' Google Trends (2004-2015) vs. forecasts (2005-2020) in Mississippi.

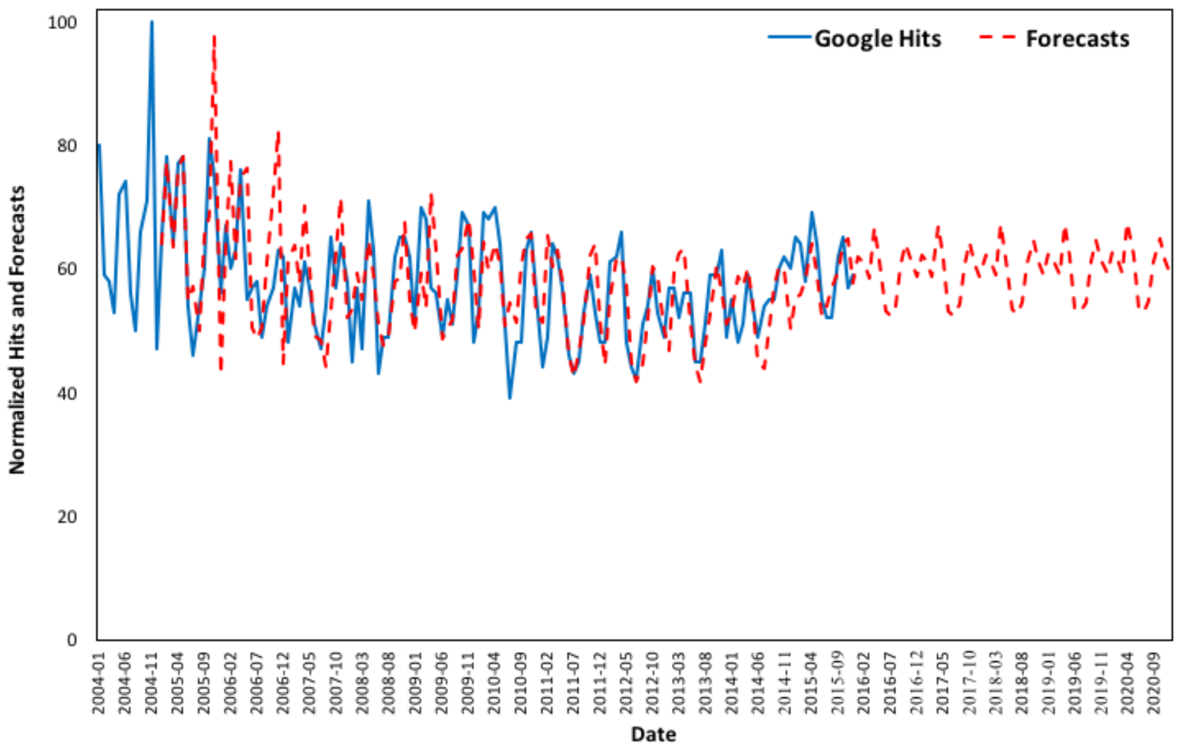

Figure B26. 'Asthma' Google Trends (2004-2015) vs. forecasts (2005-2020) in Missouri.

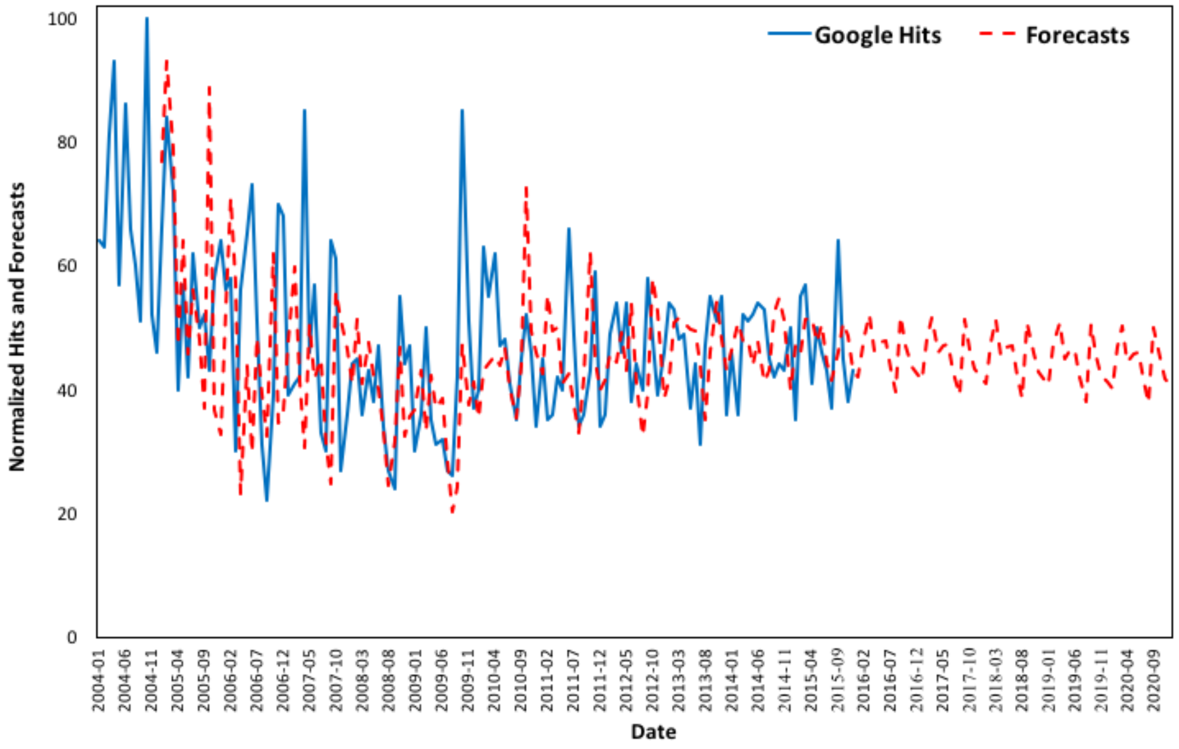

Figure B27. 'Asthma' Google Trends (2004-2015) vs. forecasts (2005-2020) in Montana.

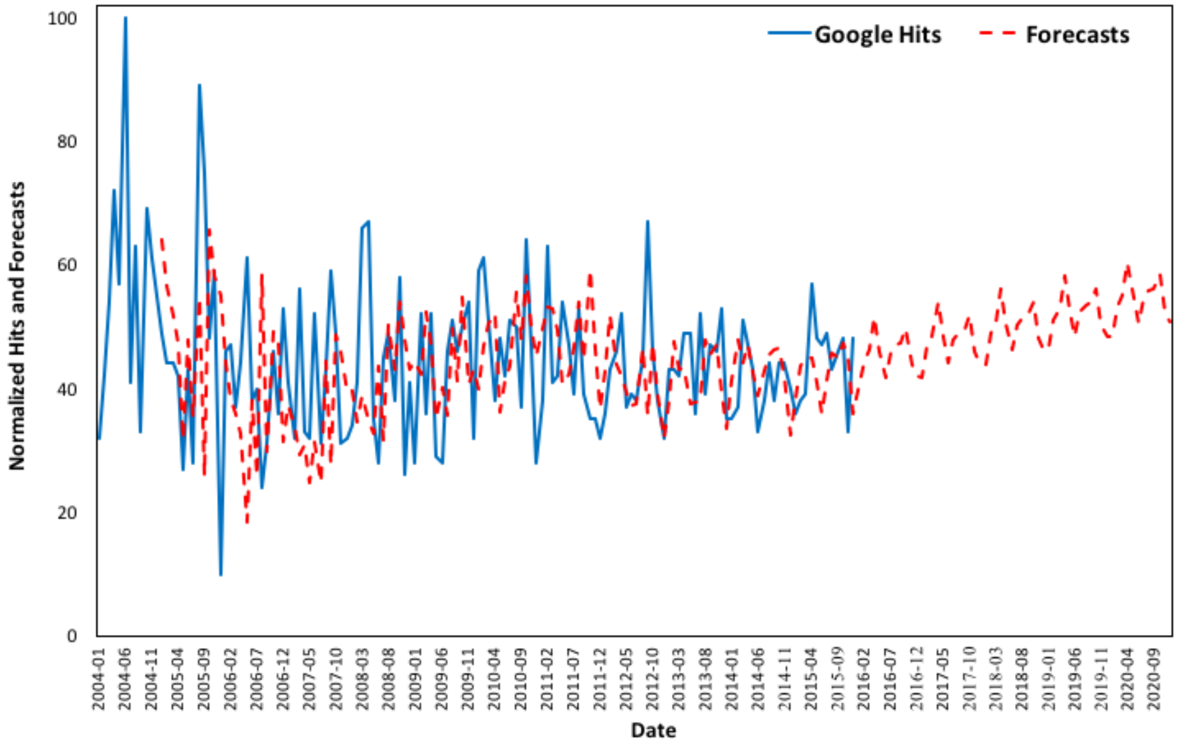

Figure B28. 'Asthma' Google Trends (2004-2015) vs. forecasts (2005-2020) in Nebraska.

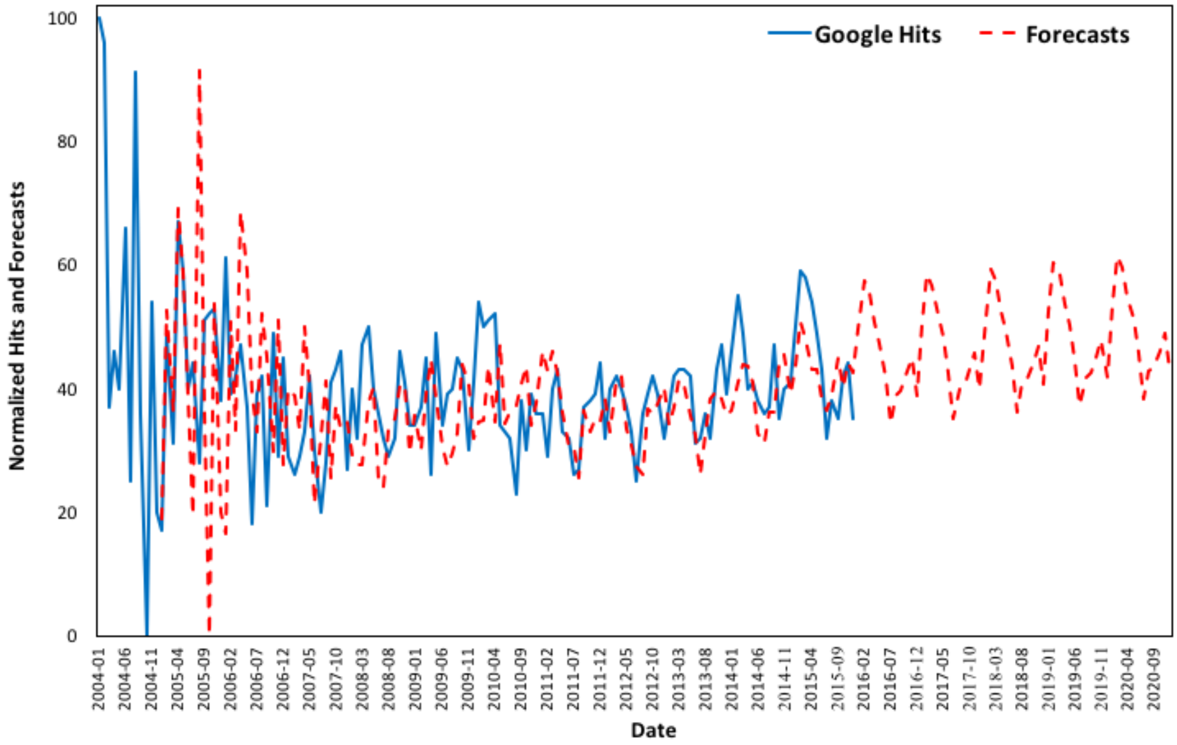

Figure B29. 'Asthma' Google Trends (2004-2015) vs. forecasts (2005-2020) in Nevada.

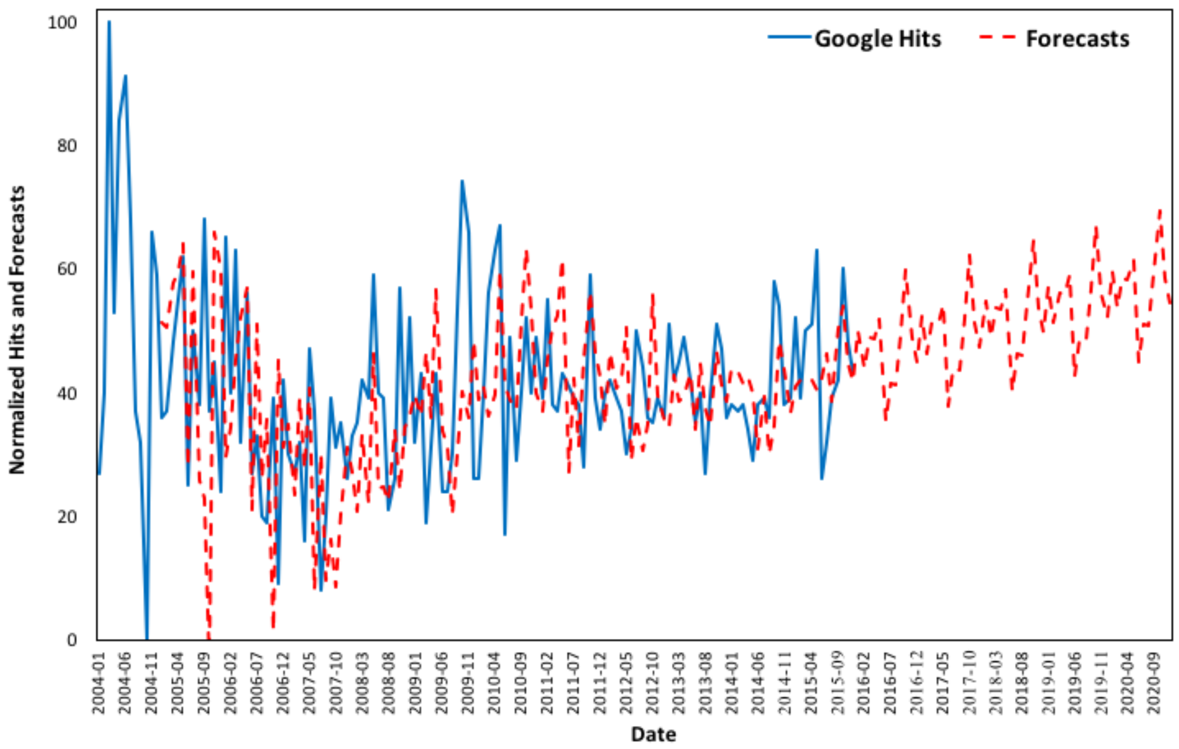

Figure B30. 'Asthma' Google Trends (2004-2015) vs. forecasts (2005-2020) in New Hampshire.

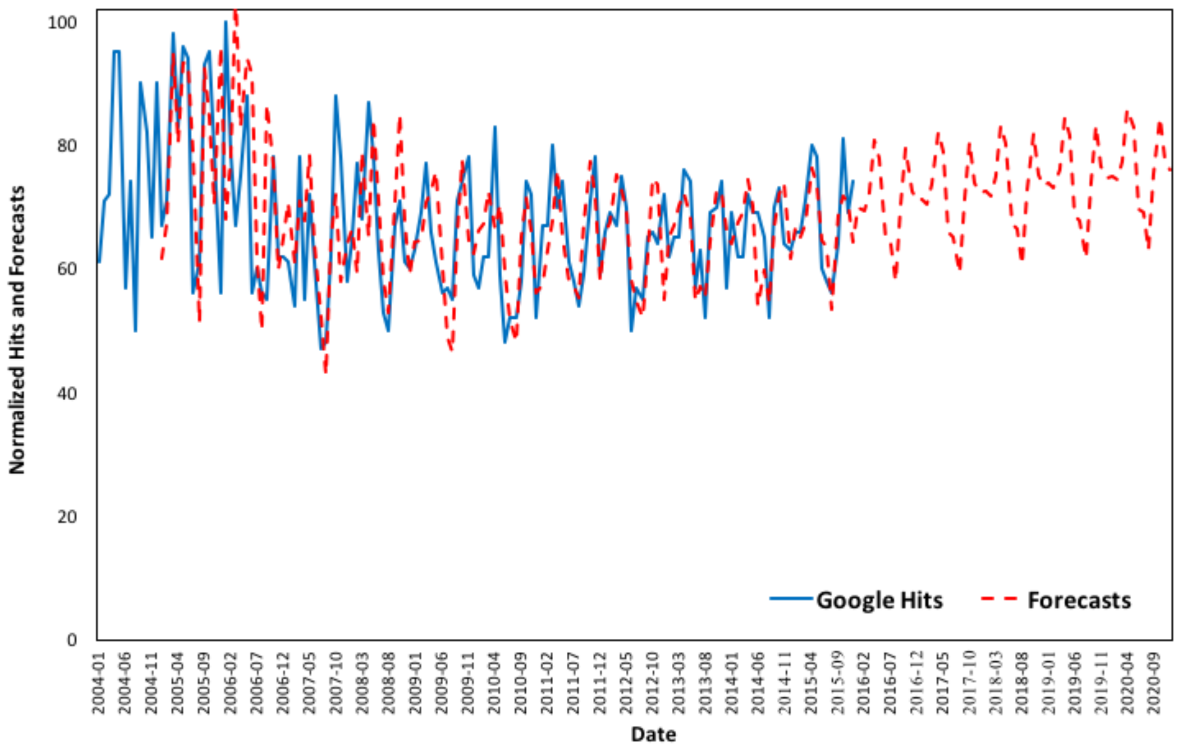

Figure B31. 'Asthma' Google Trends (2004-2015) vs. forecasts (2005-2020) in New Jersey.

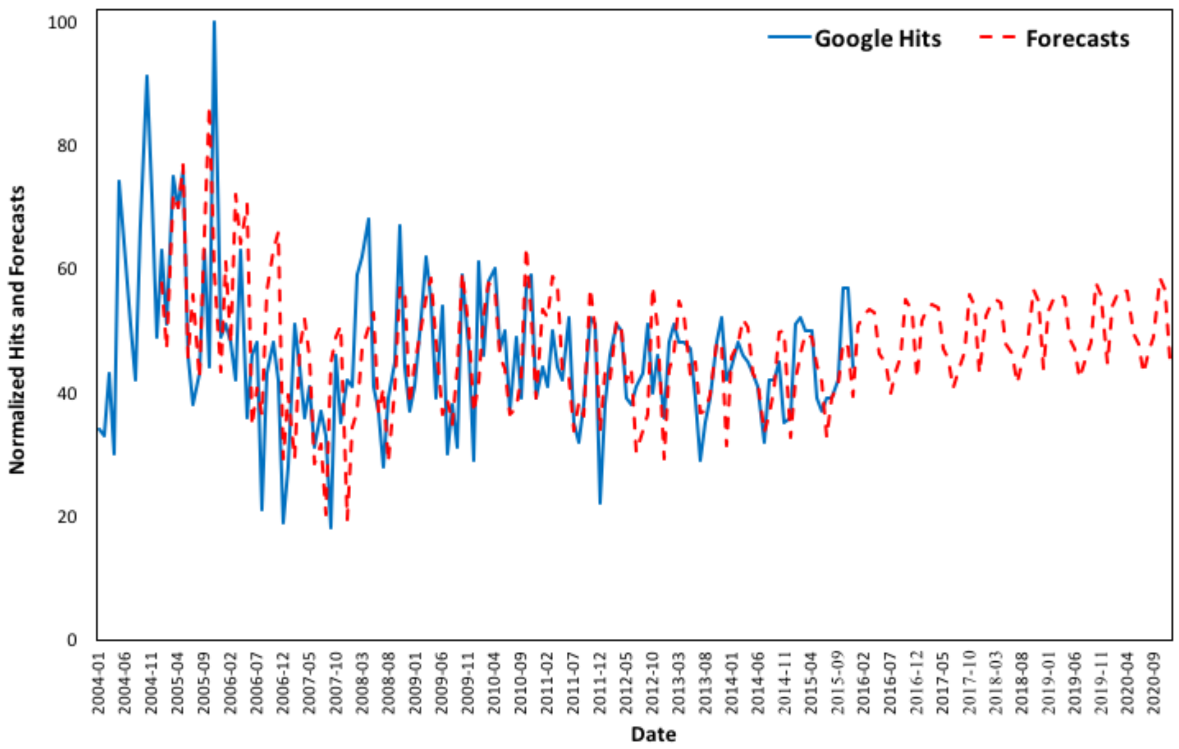

Figure B32. 'Asthma' Google Trends (2004-2015) vs. forecasts (2005-2020) in New Mexico.

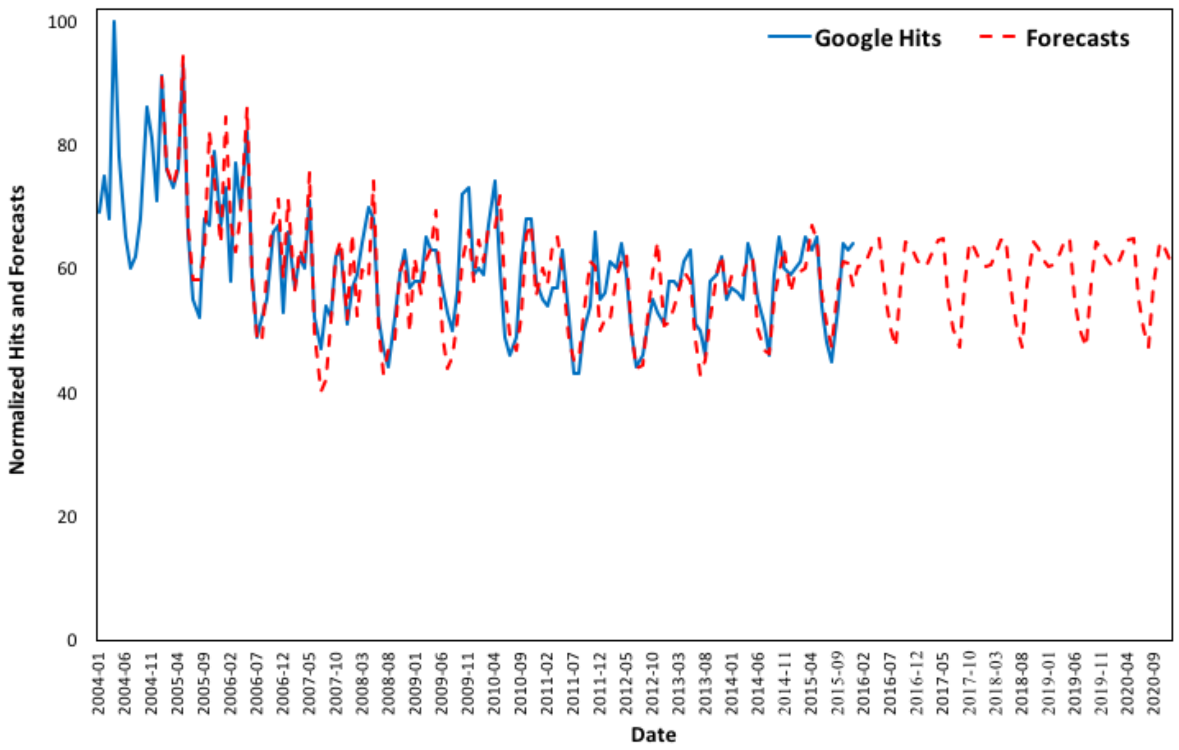

Figure B33. 'Asthma' Google Trends (2004-2015) vs. forecasts (2005-2020) in New York.

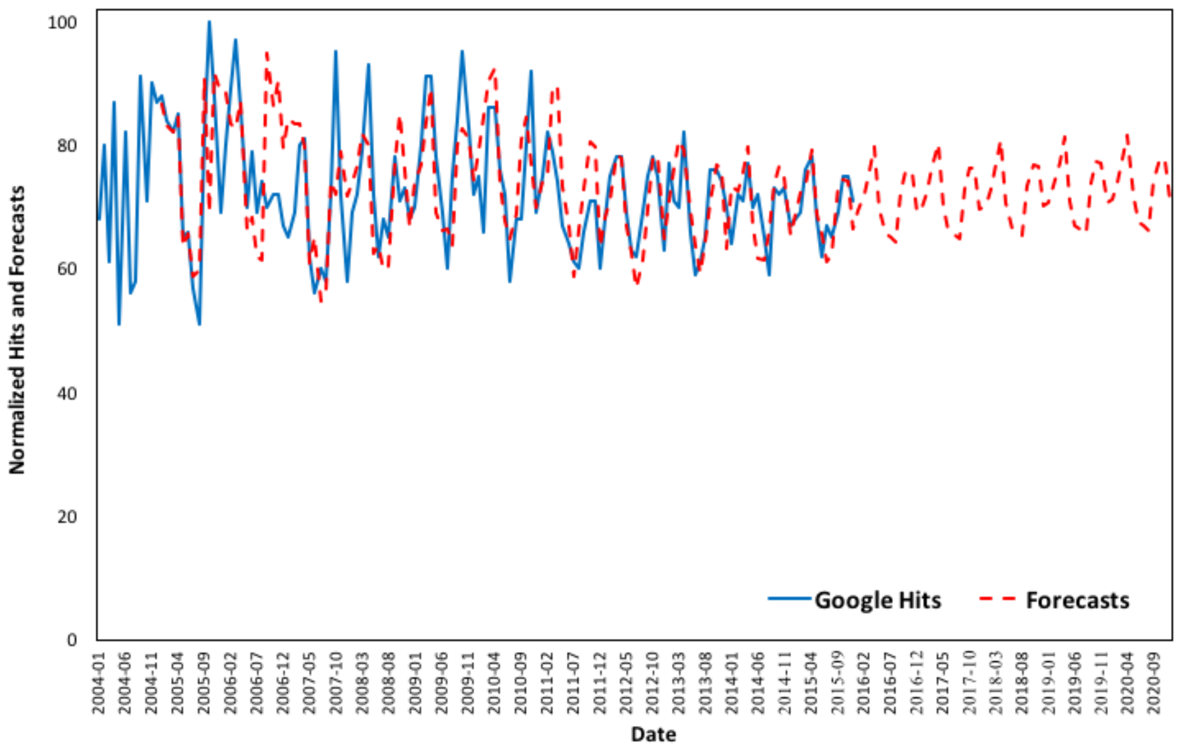

Figure B34. 'Asthma' Google Trends (2004-2015) vs. forecasts (2005-2020) in North Carolina.

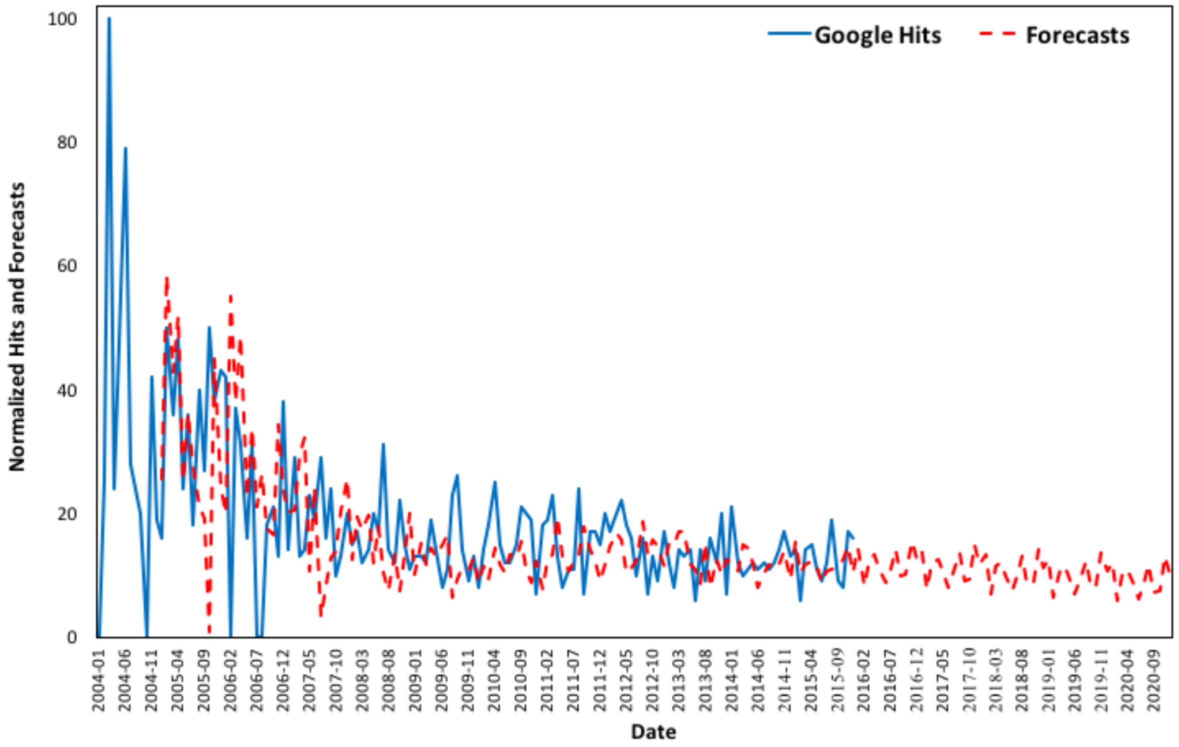

Figure B35. 'Asthma' Google Trends (2004-2015) vs. forecasts (2005-2020) in North Dakota.

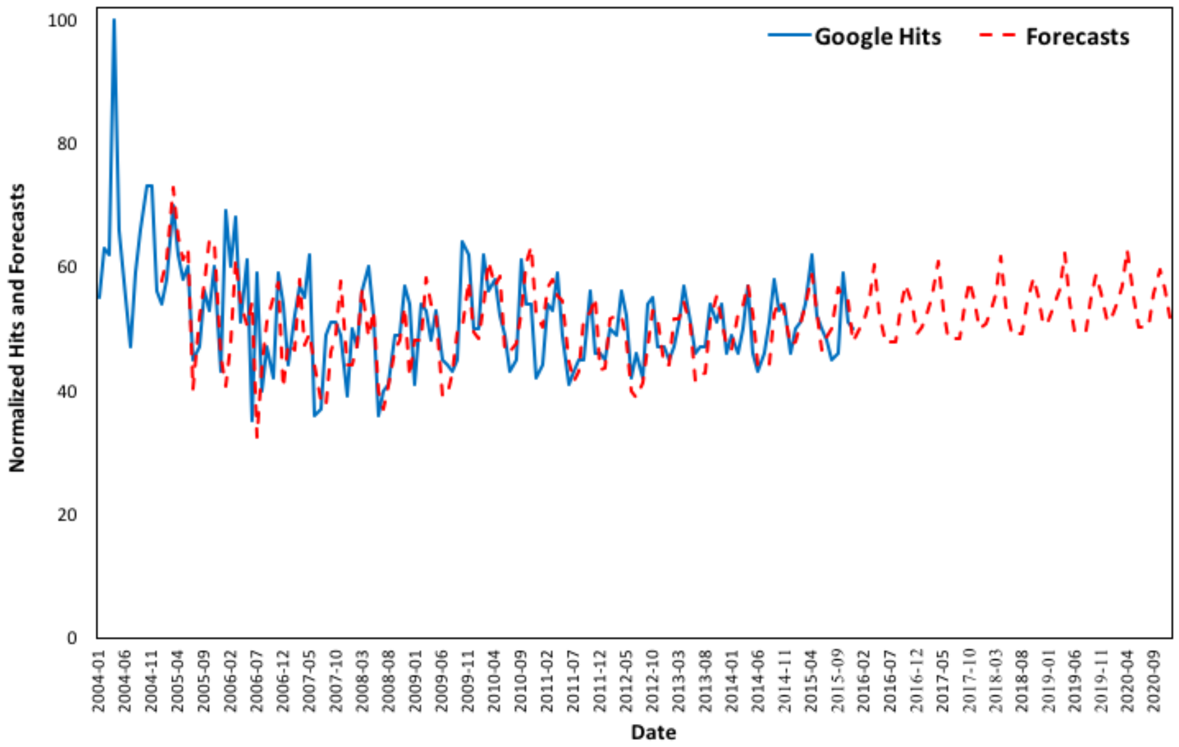

Figure B36. 'Asthma' Google Trends (2004-2015) vs. forecasts (2005-2020) in Ohio.

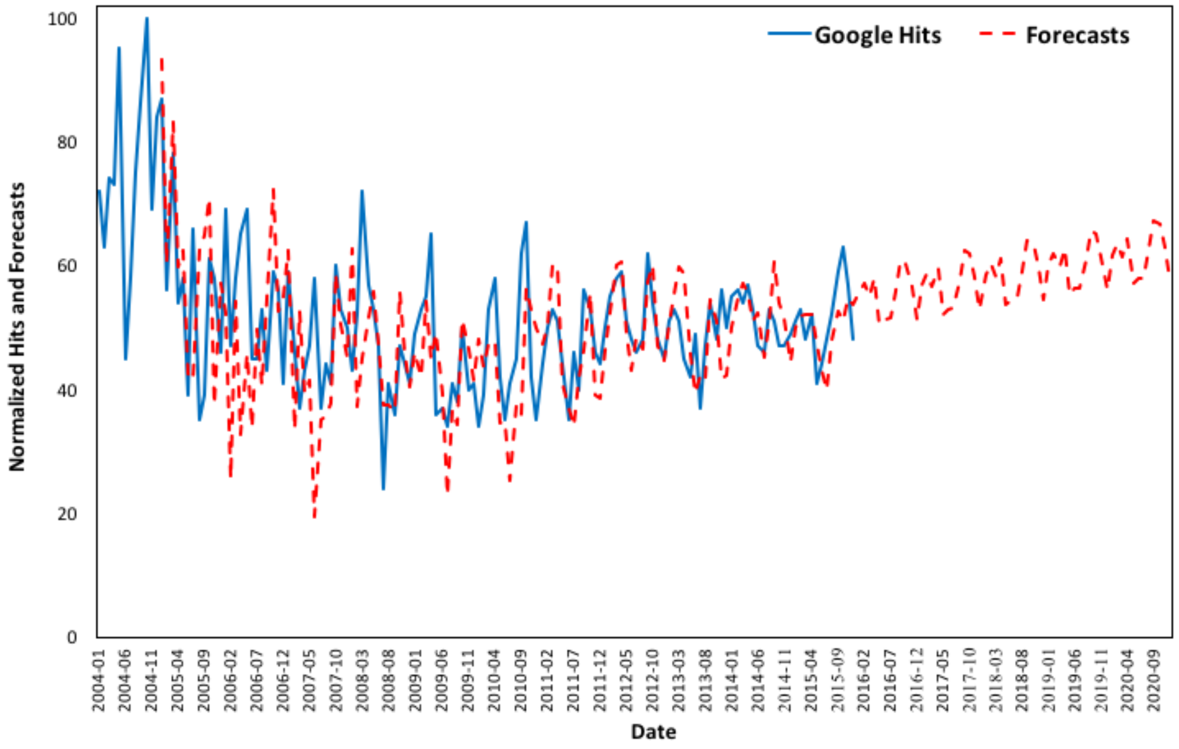

Figure B37. 'Asthma' Google Trends (2004-2015) vs. forecasts (2005-2020) in Oklahoma.

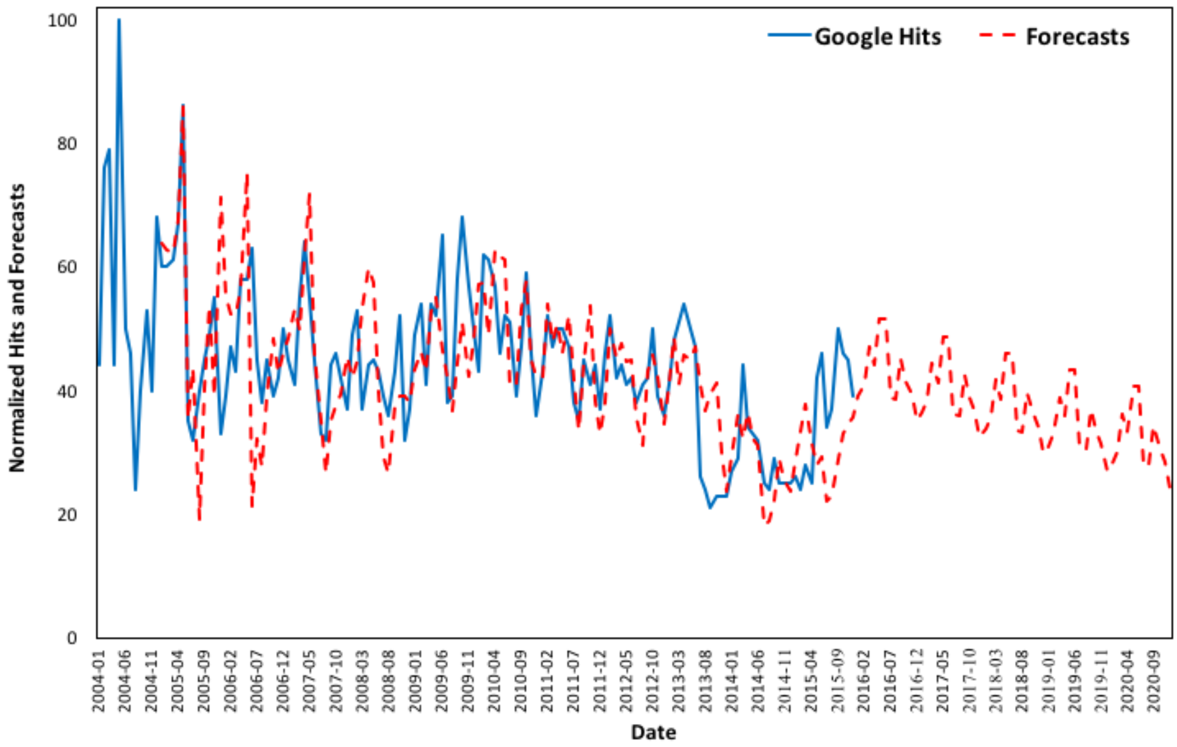

Figure B38. 'Asthma' Google Trends (2004-2015) vs. forecasts (2005-2020) in Oregon.

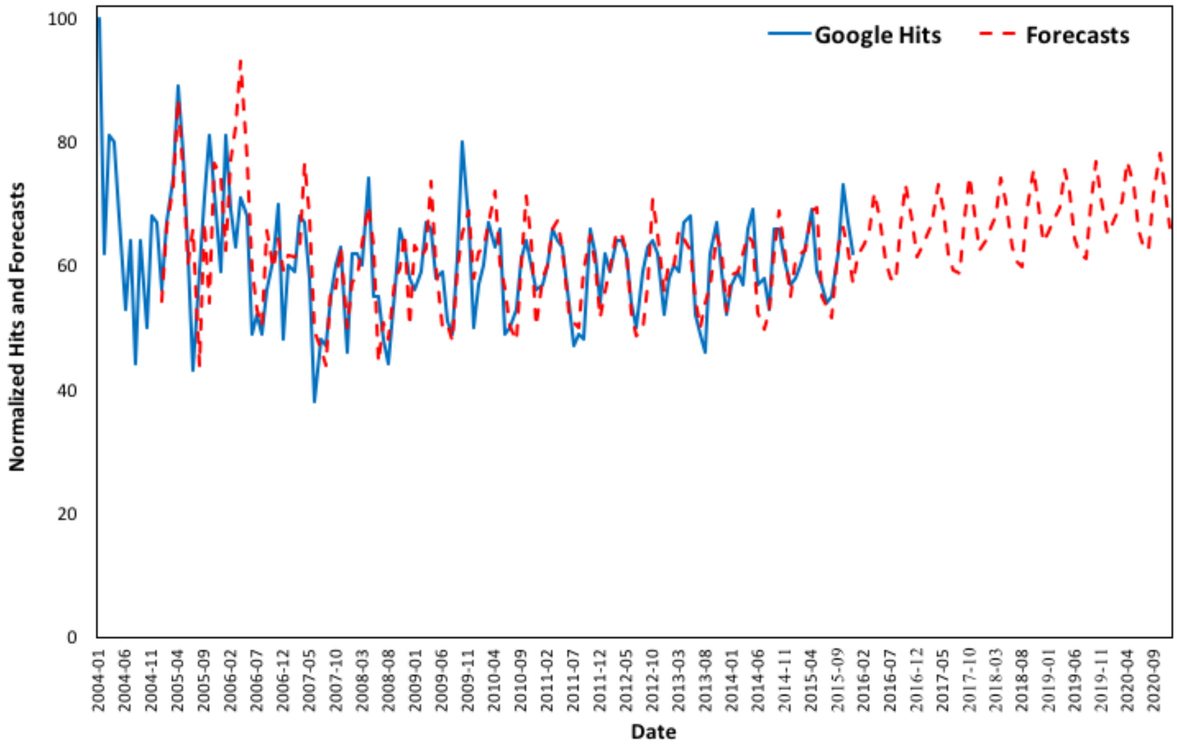

Figure B39. 'Asthma' Google Trends (2004-2015) vs. forecasts (2005-2020) in Pennsylvania.

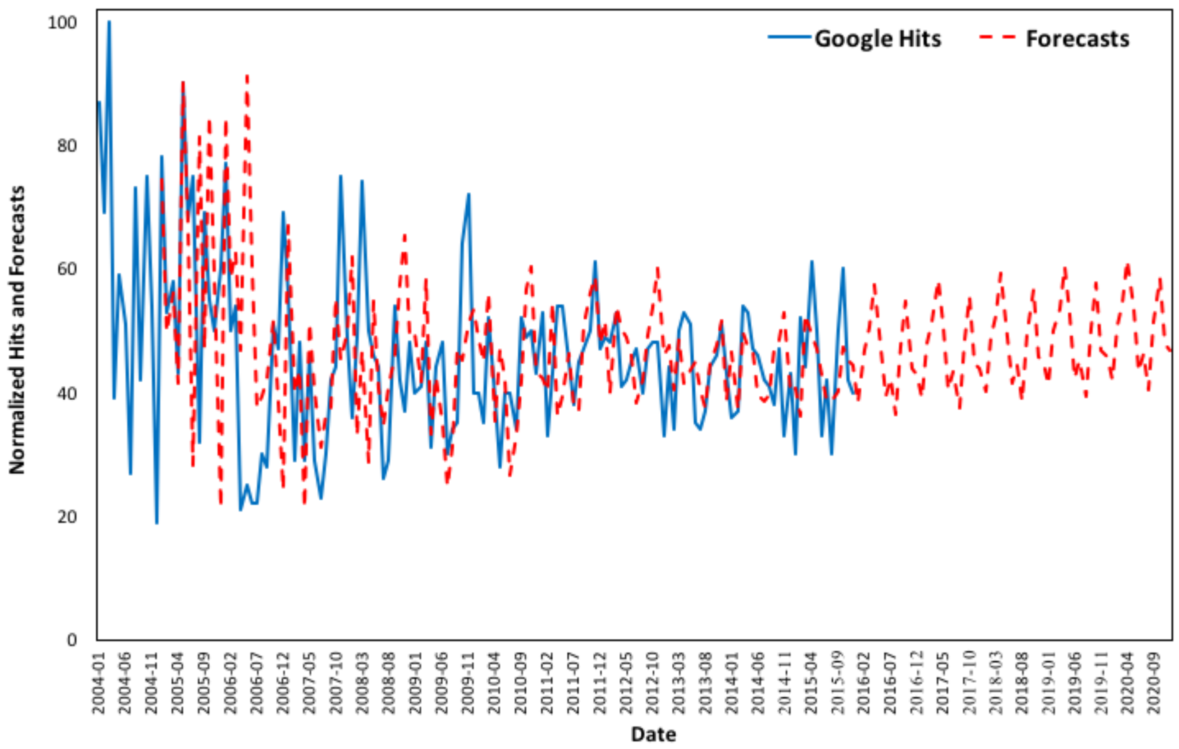

Figure B40. 'Asthma' Google Trends (2004-2015) vs. forecasts (2005-2020) in Rhode Island.

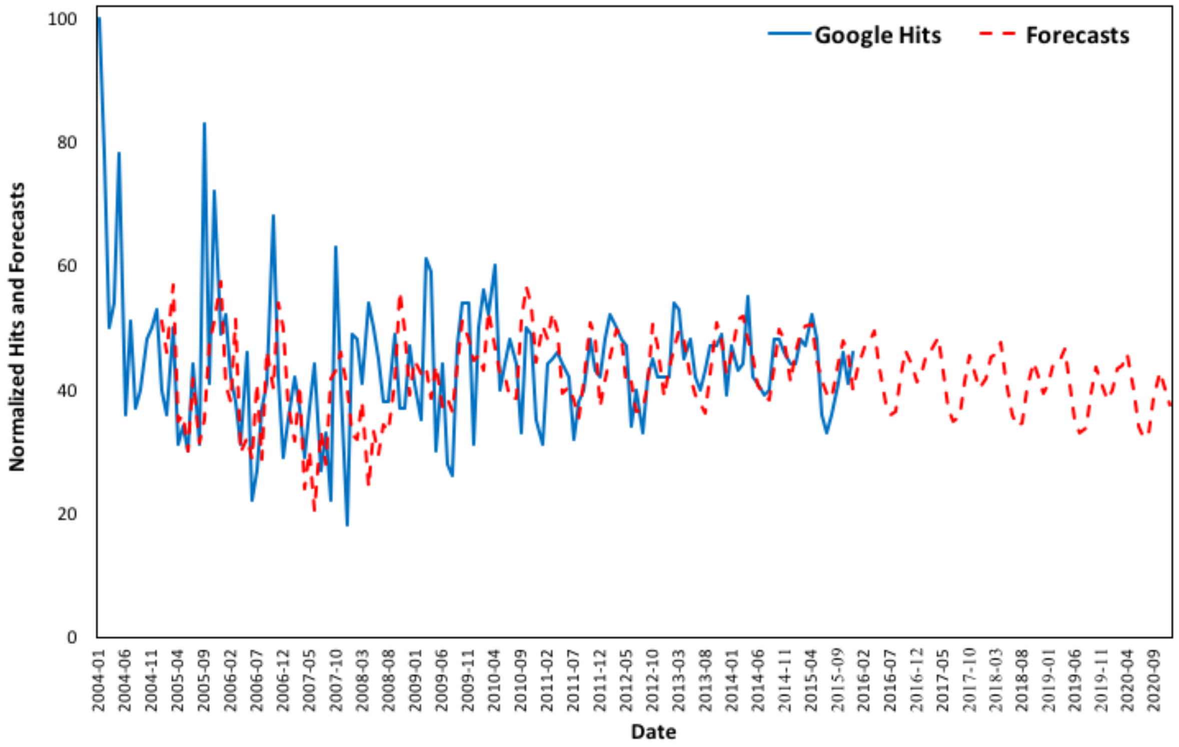

Figure B41. 'Asthma' Google Trends (2004-2015) vs. forecasts (2005-2020) in South Carolina.

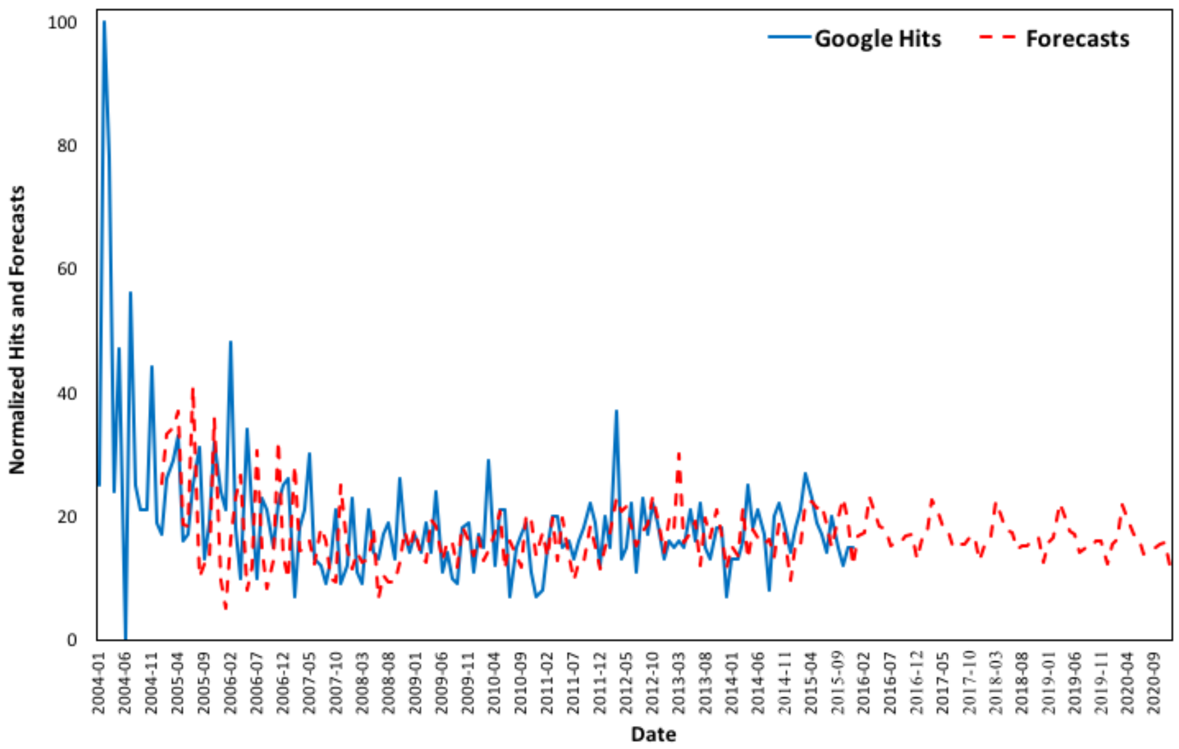

Figure B42. 'Asthma' Google Trends (2004-2015) vs. forecasts (2005-2020) in South Dakota.

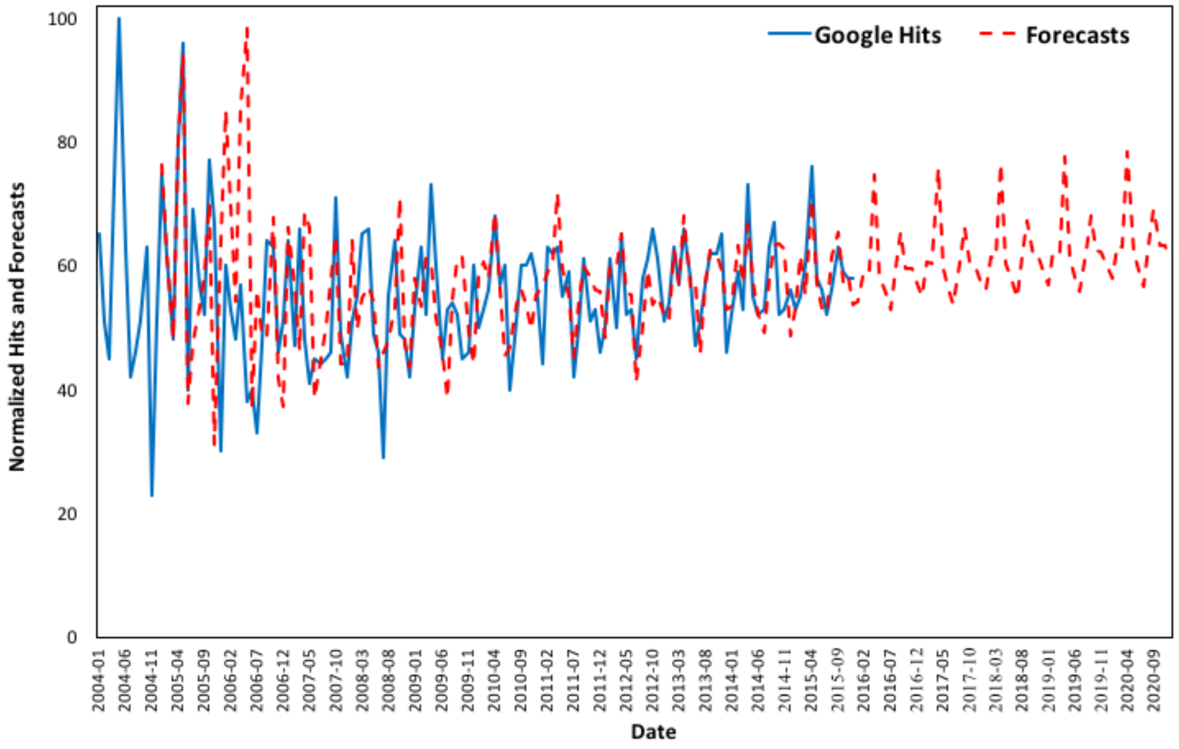

Figure B43. 'Asthma' Google Trends (2004-2015) vs. forecasts (2005-2020) in Tennessee.

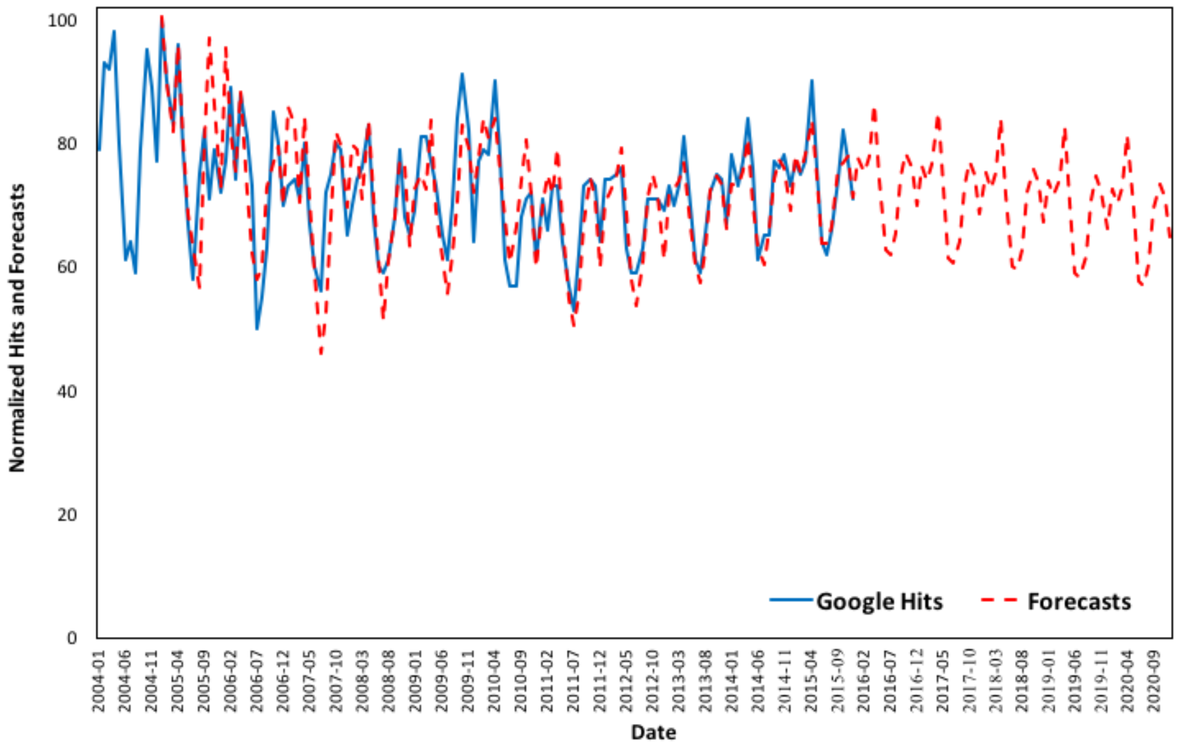

Figure B44. 'Asthma' Google Trends (2004-2015) vs. forecasts (2005-2020) in Texas.

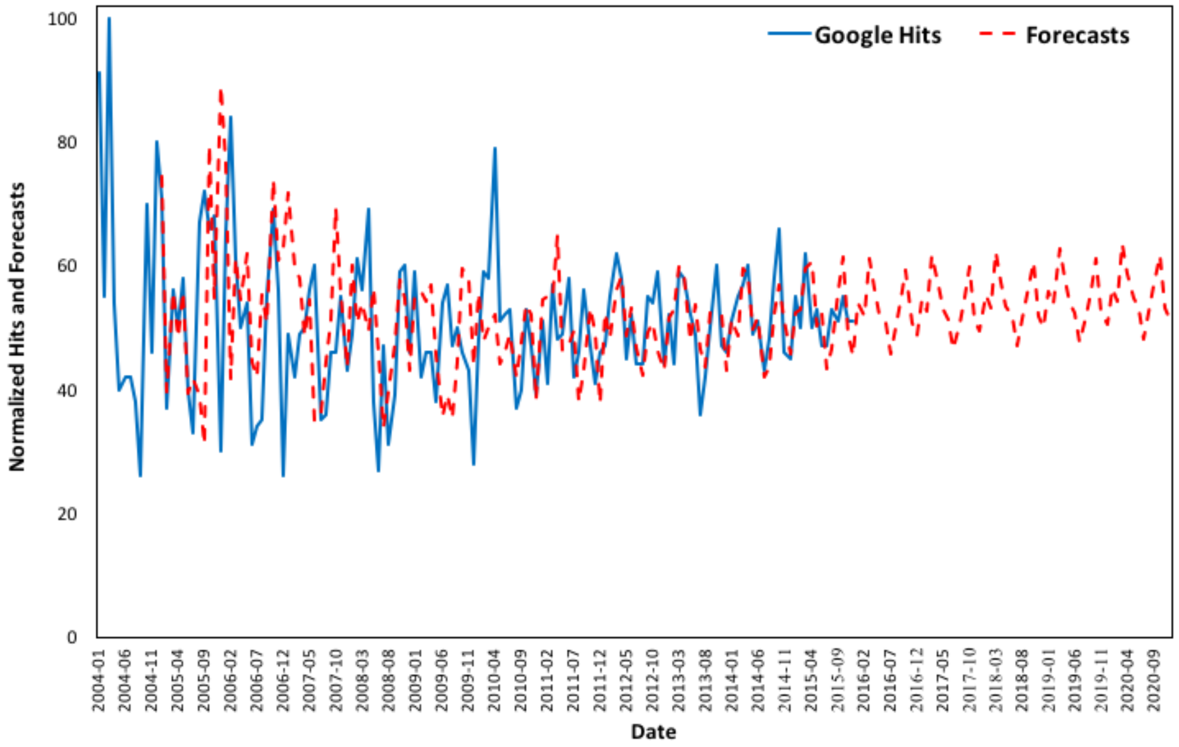

Figure B45. 'Asthma' Google Trends (2004-2015) vs. forecasts (2005-2020) in Utah.

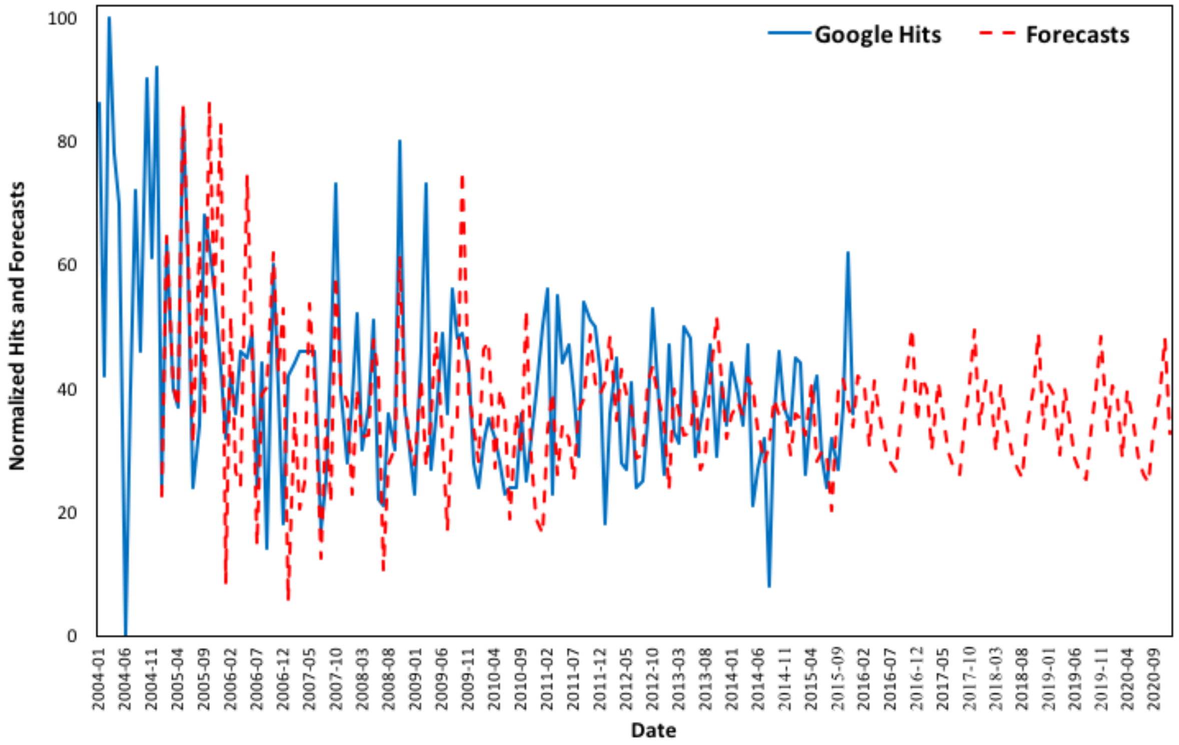

Figure B46. 'Asthma' Google Trends (2004-2015) vs. forecasts (2005-2020) in Vermont.

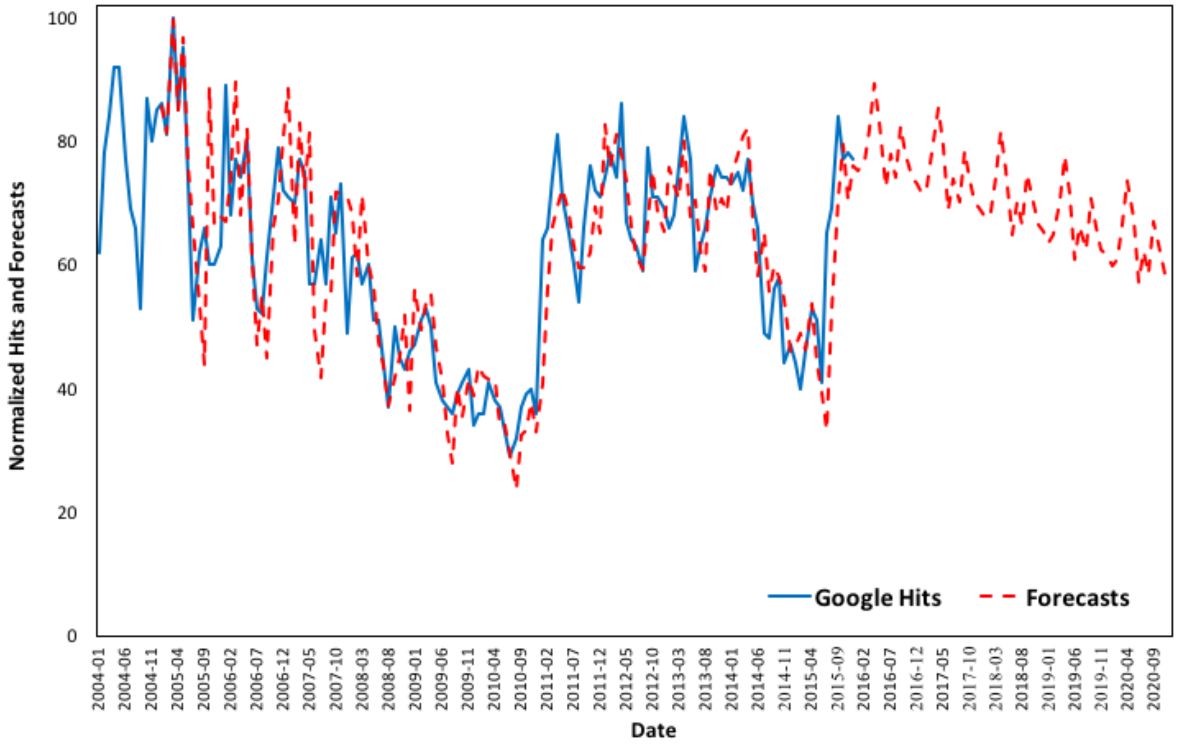

Figure B47. 'Asthma' Google Trends (2004-2015) vs. forecasts (2005-2020) in Virginia.

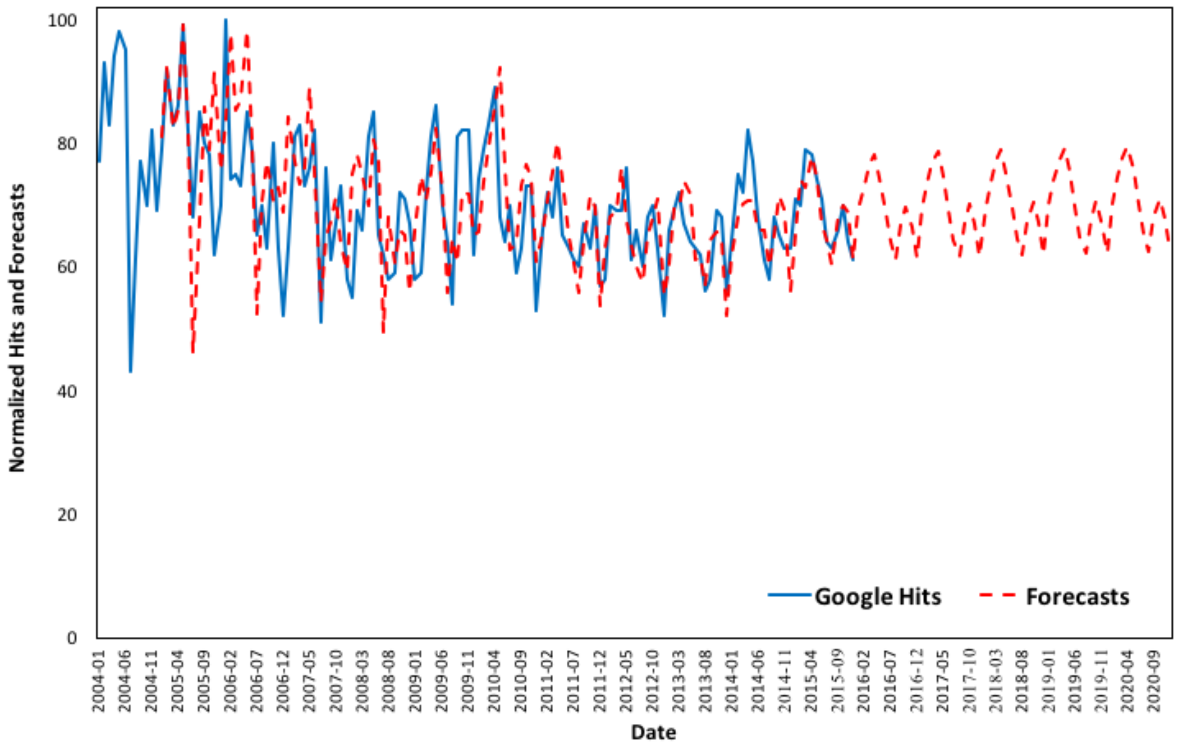

Figure B48. 'Asthma' Google Trends (2004-2015) vs. forecasts (2005-2020) in Washington.

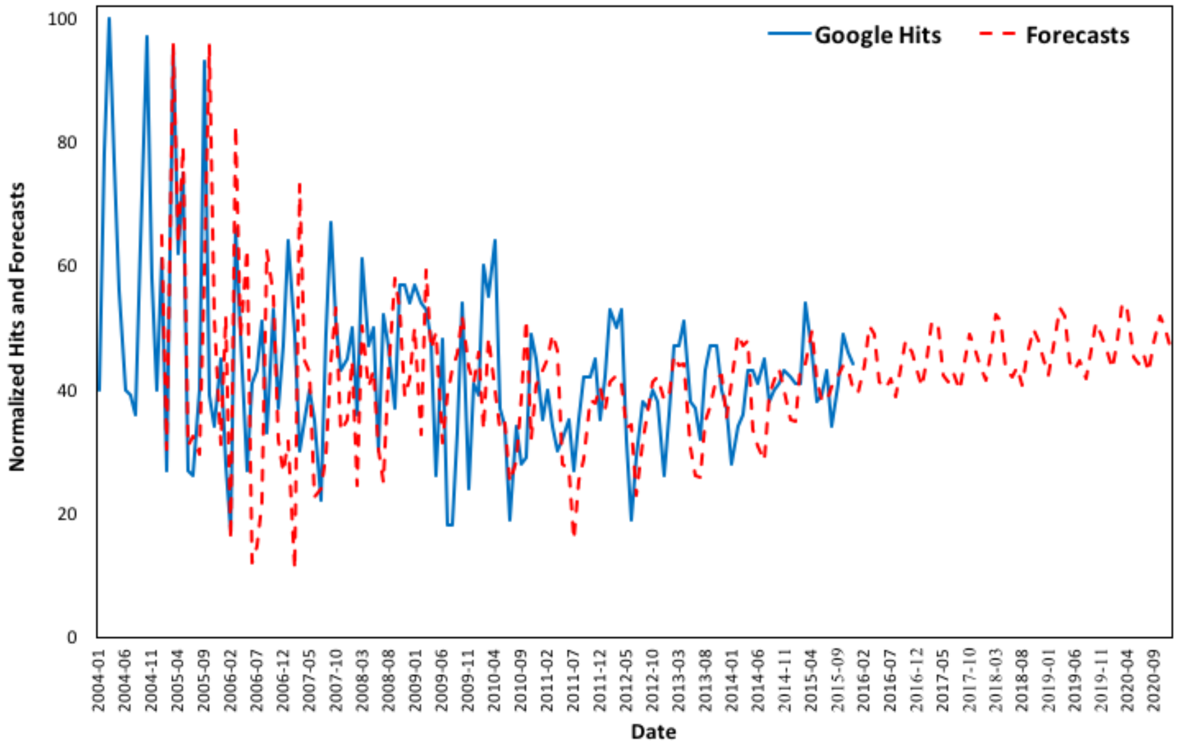

Figure B49. 'Asthma' Google Trends (2004-2015) vs. forecasts (2005-2020) in West Virginia.

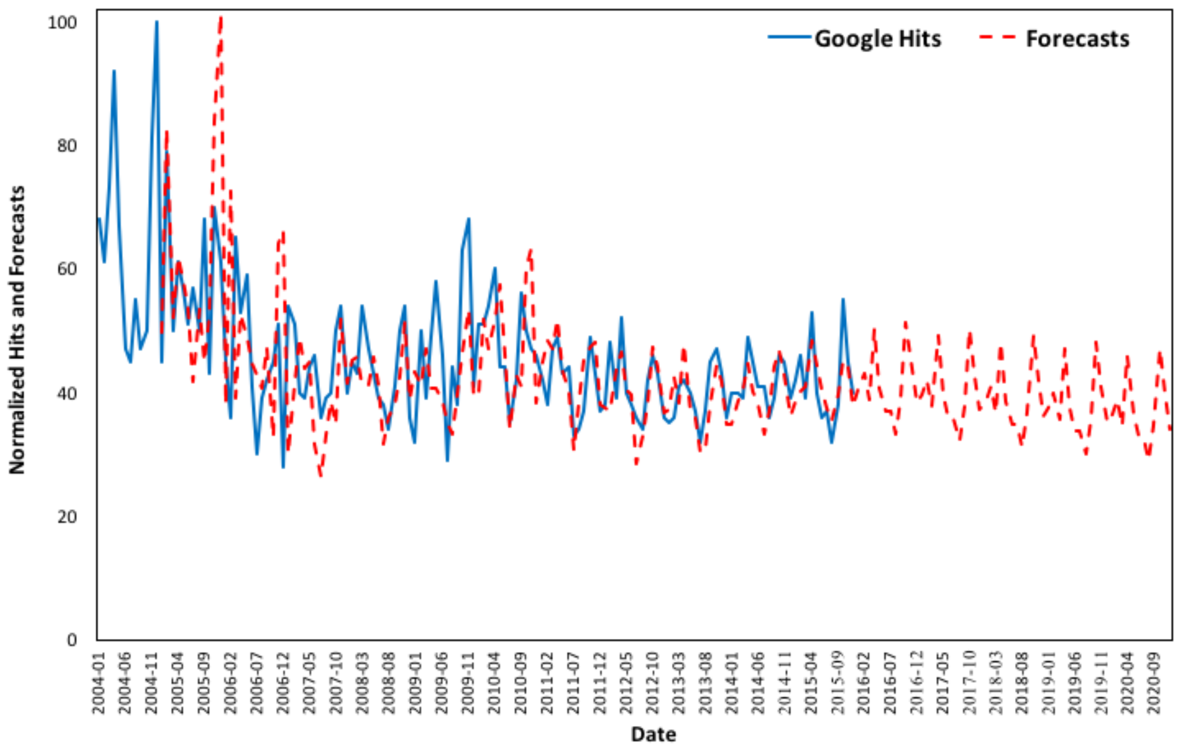

Figure B50. 'Asthma' Google Trends (2004-2015) vs. forecasts (2005-2020) in Wisconsin.

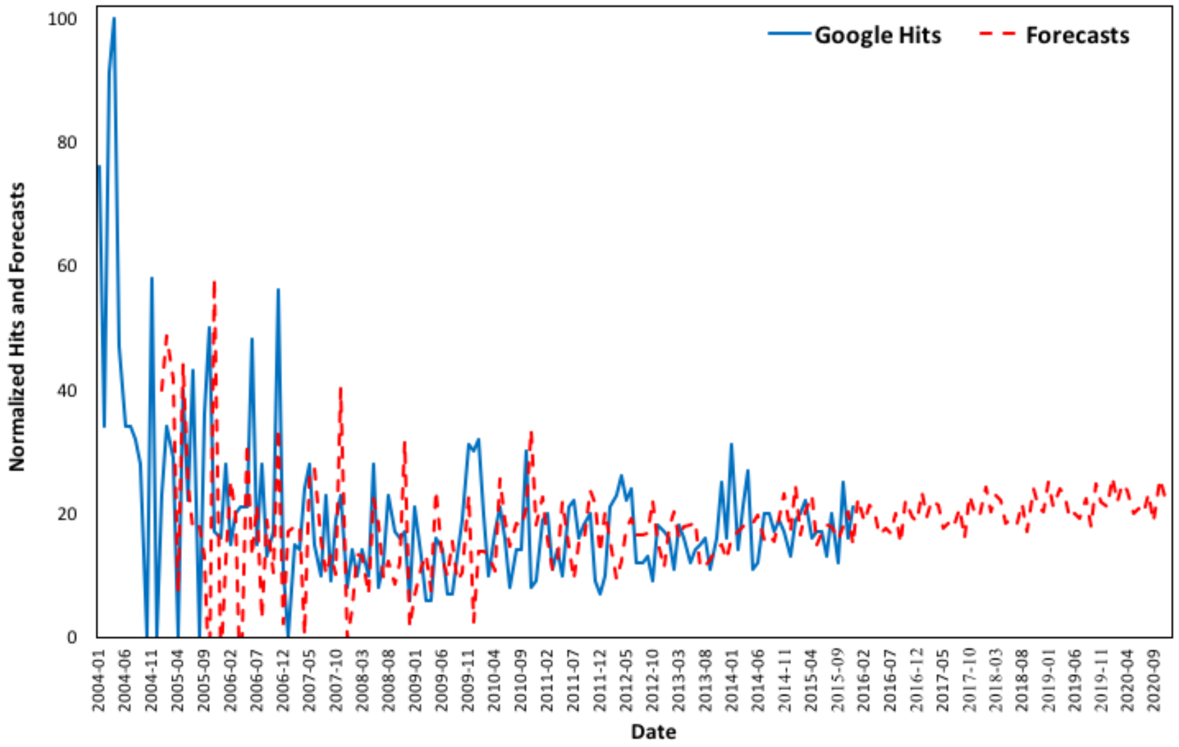

Figure B51. 'Asthma' Google Trends (2004-2015) vs. forecasts (2005-2020) in Wyoming
